# Antibacterial gas therapy: Strategies, advances, and prospects

**DOI:** 10.1016/j.bioactmat.2022.10.008

**Published:** 2022-11-11

**Authors:** Tian-Yu Wang, Xiao-Yu Zhu, Fu-Gen Wu

**Affiliations:** State Key Laboratory of Bioelectronics, School of Biological Science and Medical Engineering, Southeast University, 2 Sipailou Road, Nanjing, 210096, PR China

**Keywords:** Antibacterial, Antibiofilm, Gas therapy, Gas donor, Nitric oxide

## Abstract

One of the challenges posed by current antibacterial therapy is that the expanded and massive use of antibiotics endows bacteria with the ability to resist almost all kinds of antibiotics. Therefore, developing alternative strategies for efficient antibacterial treatment is urgently needed. Antibacterial gas therapy has attracted much attention in the past decade. Nitric oxide (NO), carbon monoxide (CO), sulfur dioxide (SO_2_), hydrogen sulfide (H_2_S), and hydrogen (H_2_) are not only known as endogenous signaling molecules, but also play critical roles in many pathological processes. These gases are considered as attractive bactericidal agents because they are able to kill bacteria, disperse biofilms, and promote bacteria-infected wound healing while avoiding resistance. In this review, we discuss the bactericidal properties of these gases, as well as the recent advances of gas-involving systems in antibacterial, antibiofilm, and wound treatment applications. Moreover, we summarize various gas donors utilized in antibacterial treatment. We hope this review will shed new light on the future design and applications of advanced antibacterial gas therapy.

## Highlights

• All kinds of gases with antibacterial effect are included in this review.
• The general antibacterial mechanisms of different gases are introduced.
• The fabrication and therapeutic effects of gas-releasing systems are described.
• The limitations and future directions of antibacterial gas therapy are discussed.

## Graphical abstract

**Figure:**
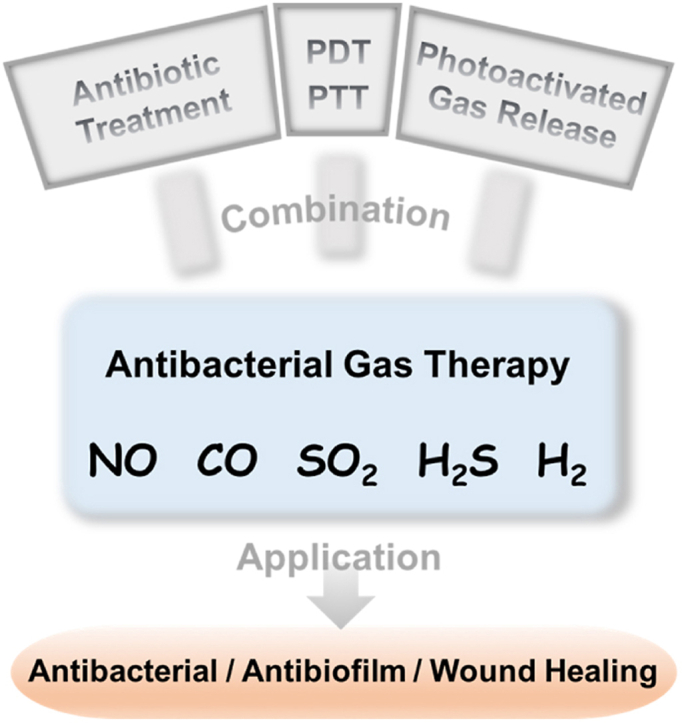

## 1 Introduction

Since Alexander Fleming reported the antibacterial activity of penicillin in 1928, antibiotics have been extensively applied in antibacterial treatment and have saved numerous lives. Generally, traditional antibiotics are derived from natural substances or chemically synthesized, and they can selectively inhibit or eliminate bacteria through inhibiting protein synthesis, DNA replication and repair, and cell-wall turnover [1]. However, with the extensive use of antibiotics, bacteria have achieved antibiotic resistance to almost all kinds of traditional antibiotics by de novo mutation or acquiring genes from other organisms [2]. What is more, clusters of bacteria can embed into a self-secreted extracellular polymeric substance matrix to form a three-dimensional biofilm, leading to higher resistance to antibiotics [3,4]. Therefore, it is urgent to develop highly effective antibiotic alternatives with properties of small dosage, long duration of efficacy, and excellent biocompatibility to mitigate the increased antibiotic resistance [5,6].

So far, many researchers including our group have developed various antimicrobial materials such as natural antimicrobial materials [7,8], carbon dot-based materials [[9], [10], [11], [12], [13], [14], [15]], graphene and its derivatives [[16], [17], [18], [19]], silicon-containing materials [[20], [21], [22], [23], [24]], metal-containing materials [[25], [26], [27], [28], [29], [30], [31]], metal−organic-frameworks (MOFs) [[32], [33], [34]], hydrogels [[35], [36], [37]], polymeric materials [[38], [39], [40]], etc., each with their own advantages and shortcomings. For instance, most metal-ion and semiconductor materials are hard to induce bacterial resistance [41]. In addition, organic antimicrobial and metal-ion materials have a short duration of efficacy and will rapidly release active species [42,43], and the limited light-absorption capability and insufficient catalytic activity of photocatalytic antimicrobial materials also lead to low antibacterial efficacy [44].

**Scheme 1:**
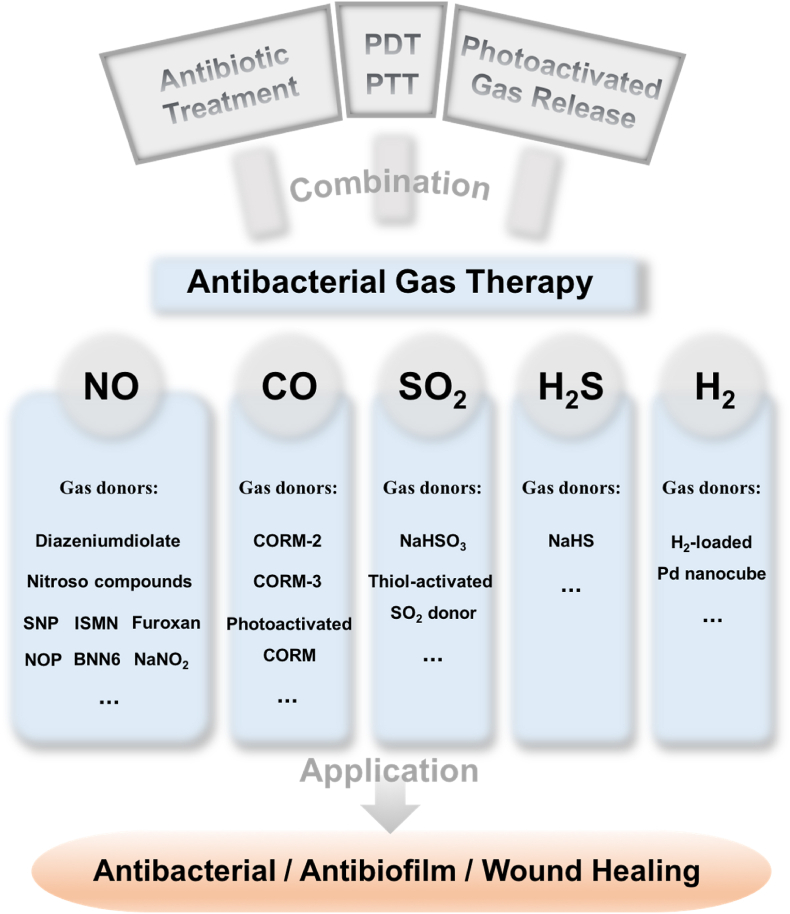
Typical gases and corresponding gas donors for antibacterial gas therapy and gas-involved combination therapies. Abbreviations: SNP: sodium nitroprusside; ISMN: isosorbide mononitrate; NOP: *N*-(3-aminopropyl)-3-(trifluoromethyl)-4-nitrobenzenamine; BNN6: *N*,*N*′-di-*sec*-butyl-*N*,*N*′-dinitroso-1,4-phenylenediamine.

In recent years, physiologically significant gases including nitric oxide (NO), carbon monoxide (CO), sulfur dioxide (SO_2_), hydrogen sulfide (H_2_S), and hydrogen (H_2_) have been developed as novel therapeutics for antibacterial applications. Their ultralow molecular weights allow them to freely diffuse into biological membranes to exert their antibacterial effects inside bacterial cells or biofilms. Furthermore, these gases have long been recognized as significant compounds produced in mammalian and bacterial cells in low amounts, which mediate important physiological processes such as regulating cardiac function and blood vessels or acting as multifunctional cellular messengers [[45], [46], [47]]. Among these gases, NO is the most extensively studied endogenously produced gaseous molecule, which plays a critical role against infection. It acts as a signaling molecule to promote the activity of immune cells at low concentrations, while it can covalently bind to DNA, proteins, or lipids of pathogens to kill them at high concentrations [48]. Therefore, few bacteria are able to escape the antibacterial effect of NO. CO is considered to bind to the oxidase active site of bacterial respiratory chain, thus hindering respiration to kill bacteria [49]. SO_2_ has been utilized as an antimicrobial agent in winemaking and as an antioxidant and preservative in the food and pharmaceutical industries for a long time [50]. At high concentrations, it can damage biomacromolecules, which exhibits enormous potential in antibacterial applications [51]. In addition, several studies have also reported the antibacterial potency of H_2_S [[52], [53], [54], [55], [56], [57], [58], [59]] and H_2_ [60,61]. Moreover, integrating gas therapy with the common antibacterial strategies such as photodynamic therapy (PDT), photothermal therapy (PTT), or antibiotic treatment can potentially improve their therapeutic efficacy. Direct delivery of exogenous gaseous molecules is the simplest route to administer gases. However, such a direct administration approach may encounter problems when applied in real physiological conditions because of the toxicity and uncontrollable nature of these gases. Once high concentrations of these gases diffuse into air, they will inevitably cause damage to respiratory systems and even to normal tissues [62]. Hence, it is necessary to develop gas delivery systems to release gaseous molecules in a controlled and targeted manner. This review mainly focuses on the recent advances in gas-releasing therapies against different kinds of bacteria, biofilms, and wound infections (Scheme 1). We put emphasis on the controllable release to realize the gas therapy alone or the combination treatment together with other therapies, and highlight their antibacterial efficacies and application prospects.

## 2 NO therapy

NO is an endogenously produced gaseous molecule implicated in diverse physiological processes, and it plays a dual role as both a critical molecule in signaling and a cytotoxic antibacterial agent [63]. In general, three kinds of NO synthase (NOS) isoforms (endothelial, neuronal, and inducible NOS) can catalyze l-arginine (L-Arg) into NO *in vivo* [64]. Endothelial and neuronal NOS can produce NO at low concentrations to regulate physiological processes such as angiogenesis, vasodilation, and neurotransmission [65], while inducible NOS can produce NO at higher concentrations in macrophages and neutrophils to respond to foreign pathogens [66]. NO can react with superoxide that was endogenously derived from the respiration process of bacteria to generate NO radical (NO•), peroxynitrite (ONOO^−^), and dinitrogen trioxide (N_2_O_3_), which exhibit antibacterial effects on various bacteria by causing the oxidative or nitrosative stress such as DNA deamination and lipid peroxidation [67]. Moreover, exogenous NO also exhibits antibacterial activity. For example, a recent clinical trial reported that a patient with pulmonary *Mycobacterium abscessus* was treated with adjunctive intermittent NO, and improvements in lung function were observed [68].

Besides being an important component of the body's innate immune response, NO is also recognized as a potent regulator to disperse biofilms. Most bacterial infections are related to the biofilm formation. Biofilms are protected by extracellular polysaccharide matrices which either impede the immune response or diminish antibiotic efficacy [69]. However, NO can regulate the cyclic dimeric guanosine monophosphate (c-di-GMP, a signaling molecule involved in the generation and maintenance of the biofilm extracellular polysaccharide matrix) level and turn biofilm cells into free-swimming cells, thus leading to biofilm dispersion and increasing the susceptibility of bacteria to antibiotics [67,70]. For example, Howlin and coworkers demonstrated that submicromolar NO concentrations effectively treated *Pseudomonas aeruginosa* (*P*. *aeruginosa*) infection in cystic fibrosis and exhibited a significant decrease in *P*. *aeruginosa* biofilm tolerance to tobramycin and tobramycin combined with ceftazidime [71]. Huang et al. demonstrated that NO pretreatment significantly increased ofloxacin efficacy by reducing exopolysaccharides in biofilms [72].

Although NO is proved to be efficient in killing bacteria and disrupting biofilms, direct delivery of gaseous NO to bacterially infected sites is therapeutically intractable [67]. In addition, application of exogenously delivered gaseous NO in antibacterial treatment is usually impeded due to its extremely short biological lifetime [73]. Hence, diverse NO- or NO donor-containing systems and NO-producing approaches (e.g., bacteria-mediated NO generation) have been developed for effective NO delivery (Table 1). Among these systems, several light-triggerable NO-releasing systems have been designed for precise delivery and controlled release of NO to infected sites. In addition, NO therapy is also combined with antibiotics, PDT, PTT, or other gases to exert more satisfactory antibacterial activity.

**Table 1:** NO-containing systems for antibacterial therapy.

Gas donor	System	Combined therapy or treatment	Other materials	Ref.
Diazeniumdiolate	Amphiphilic *N*-diazeniumdiolate-functionalized PAMAM conjugate	–	PAMAM, propylene oxide, and 1,2-epoxy-9-decene	[76]
	*N*-Diazeniumdiolate-modified hyperbranched PAMAM polymer	–	Hyperbranched PAMAM	[77]
	F68-BPEI-NONOate	–	Pluronic F68 and branched PEI	[78]
	*N*-Diazeniumdiolate-functionalized *β*CD derivative	–	*β*CD and *N*-(2-hydroxy ethyl) ethylene-diamine	[79]
	NO-releasing *β*CD	–	*β*CD	[80]
	*N*-Diazeniumdiolate-modified alginate	–	Alginate	[81]
	NONOate loaded POEGMA-b-PGMA NP	–	POEGMA and GMA	[82]
	PEI/NONOate-doped PLGA NP	–	PEI and PLGA	[83]
	PDA-NO HNP	–	PDA	[84]
	PROLI/NO	–	AgSD	[85]
	BORO/NO	–	Boronate ester	[86]
	CPCS-bPEI-NO	–	–	[87]
	CS-PAMAM/NONOate	–	PAMAM dendron-grafted chitosan	[88]
	CPA-CDs/NONOate	–	Chitosan-*graft*-poly(amidoamine) dendrimer	[89]
	COS–NO	–	Chitosan oligosaccharide	[90,91]
	COS/NO	–	Chitosan oligosaccharide	[92]
	COS-EA/NO	–	Ethanolamine-modified chitosan oligosaccharide	[93]
	PNBNP	–	PLGA, PLH, and PEG	[96]
	Fimbrolide−NO hybrid	–	Marine algae fimbrolide derivatives	[97]
	Superhydrophobic NO-releasing xerogel	–	Fluorinated silane/silica composite and xerogel	[114]
	NO-releasing PDA coating	–	Glass substrate, PEG, and PDA	[115]
	Nbi/NO/Cu film	–	Branched PEI, alginate, and Cu(II)	[116]
	PTMSPA- and DET3-decorated SE and PET	–	PTMSPA, DET3, SE, and PET	[126]
	Diazeniumdiolate-functionalized Ti surface	–	Ti rod, 6-aminohexyl-3-aminopropyltrimethoxysilane, and 11-aminoundecyltriethoxysilane	[127]
	Dendrimer-doped composite polyurethane fiber	–	Octyl alkyl chain- or quaternary ammonium moiety-functionalized PAMAM dendrimer	[128]
	Hyperbranched polyaminoglycoside	Kanamycin, GEN, and neomycin	*N,N*′-methylenebis(acrylamide)	[146]
	Cephalosporin-3′-diazeniumdiolate	Cephalosporin	–	[[148], [149], [150]]
	Cephalosporin-3′-diazeniumdiolate	Cephalosporin and azithromycin	–	[151]
	DEA-C3D	Tobramycin and colistin	–	[152]
	CS-PAMAM-MET/NONOate	MET	MET and CS-PAMAM	[137]
	Fe_3_O_4_@PDA@PAMAM@NONOate	PTT	Fe_3_O_4_@PDA@PAMAM	[155]
	Nitroaromatic-protected piperazine diazeniumdiolate	–	Nitroaromatic-protecting group	[169]
GSNO	SNO/GSNO-loaded porous silicon NP	–	SNO and porous silicon NP	[99]
	GSNO/Vaseline/ZnO	–	Vaseline and ZnO	[100]
	GSNO-loaded chitosan film	–	Chitosan film	[117]
	Phe-PEUs/PAN-G	–	Phe-PEU and PAN	[131]
Nitroso compounds	SNO/GSNO-loaded porous silicon NP	–	GSNO and porous silicon NP	[99]
	AgNP and *S*-nitroso-mercaptosuccinic acid-loaded alginate hydrogel	–	AgNP and alginate hydrogel	[101]
	SNO-functionalized coating	–	3-Mercapto-3-methylbutan-1-ol	[119]
	CS-PVA/NO hydrogel	–	Fe_3_O_4_, PDA, and chitosan-PVA hydrogel	[133]
	AuNC@NO	–	AuNC	[134]
	GS@PNO	GS	PEO	[135]
	Ir@PBNN-NO_2_	–	*fac*-Ir(ppy)_3_	[141]
	AuNR@MSN-SNO/LEVO nanoassembly	PTT	AuNR, MSN, and LEVO	[156]
	GNRs@mSiO_2_-SNO/ICG NP	PDT and PTT	Mesoporous silica-coated gold nanorod and ICG	[160]
	NO/CO-releasing donor	CO	3-HF	[170]
	PNOFA micelle	FA	Methacryloyl chloride and PEO	[166]
SNP	SNP	–	–	[102]
	SNP@MOF@Au-Mal	–	MIL, Au, and HOOC-PEG_5000_-Mal	[138]
	SNP@MOF-UCNP@ssPDA-Cy7/IR786s	–	ZIF-8, UCNP, ssPDA, Cy7, and IR786s	[139]
	MPSi-NP	–	MPSi	[153]
ISMN	ISMN-loaded PLGA NPs	–	PLGA	[103]
	ISMN immunoliposome	–	Egg lecithin, cholesterol, and anti-*S*. *aureus* α-toxin monoclonal antibody	[104]
	CS-ISMN	Ciprofloxacin	Chitosan gel	[147]
Furoxan	Furoxan compounds	–	–	[105]
	3-Nitro-4-phenylfuroxan	–	–	[106]
	FOTyr-AMP	–	AMP	[94]
Sodium nitrite (NaNO_2_)	Hydrogel/glass composite	–	Tetramethylorthosilicate, polyethylene glycol, glucose, and chitosan	[107,125]
	NaNO_2_	Ga^3+^	–	[108]
	AB569	–	–	[111]
	Polydimethylsiloxane planar patch device	–	Polydimethylsiloxane planar patch, copper(II) ligand, and gold coated steel mesh working electrode	[113]
	Electrochemical NO releasing catheter device	colistin, gentamicin, chloramphenicol, ciprofloxacin, tetracycline, and beta-defensin 2	Copper(II)-tri(2-pyridylmethyl)amine, NaCl, Ag/AgCl wire, and Teflon-coated Pt wire	[154]
	NO-producing probiotic patch	–	*L*. *fermentum* and glucose	[168]
Reaction between L-Arg and ROS	LIBDP	–	IBDP	[95]
	L-Arg@Hydrogel/H_2_O_2_	–	TSPBA, PVA, and H_2_O_2_	[109]
	AI-MPDA	PDT and PTT	L-Arg, ICG, and MPDA	[159]
	Ce6@Arg-ADP	PDT	Ce6 and ADP	[165]
SNAP	SNAP-loaded nanocellulose−chitosan layer	–	Nanocellulose−chitosan	[118]
	SNAP-incorporated film	–	CarboSil 20 80A	[120]
	SNAP-doped CarboSil polymer composite	–	CarboSil 20 80A	[121]
	CarboSil-SNAP composite	–	CarboSil 20 80A, PDA, and polytetrafluoroethylene	[122]
	SNAP impregnated silicone Foley catheter	–	Silicone Foley catheter	[123]
	SNAP-infused silicone Foley urinary catheter	–	Silicone Foley urinary catheter	[124]
	SNAP-PAN	–	PAN	[130]
	*α*-CD-Ce6-NO-DA	PDT	Ce6, *α*-CD, PEG-(KLAKLAK)_2_, and DA	[167]
NOP	PS-PEI/NOP nanofiber membrane	–	Electrospun polystyrene nanofiber membrane and PEI	[129]
	NOP/TMPyP and NOP/ZnPc	PDT	TMPyP, ZnPc, and electrospun polystyrene nanofiber	[163,164]
BNN6	GNS/HPDA-BNN6	–	GNS and HPDA	[140]
	Gel/GO-*β*CD-BNN6	PTT	*β*CD, GO, and GelMA/HA-DA hydrogel	[157]
	TP-Por CON@BNN6	PDT and PTT	HHTP and 5,15-bis(4-boronophenyl)-porphyrin	[161]
NO-loaded porous silicon NP	NO-loaded porous silicon NP	–	–	[98]
3,3-Bis(aminoethyl)-1-hydroxy-2-oxo-1-triazene	Chitosan-based polymeric NO	–	Glutaraldehyde-treated chitosan	[110]
NO-loaded PFO	PFO ME	–	–	[112]
RBS	RBS@UCNP@mSiO_2_@qC	–	UCNP@mSiO_2_ and qC	[132]
Nitrated aliphatic ester	Ocotillol-type triperpenoid nitrate	–	Ocotillol	[143]
Nitroxide carboxy-TEMPO	Carboxy-TEMPO and ciprofloxacin	Ciprofloxacin	–	[144]
PAN/NO	PAN/NO	Ciprofloxacin	–	[145]
NO-loaded POEGMA-*b*-PVBA-GEN	NO-loaded POEGMA-*b*-PVBA-GEN	–	POEGMA, PVBA, and GEN	[136]
RBNO	RBNO	PDT	–	[162]

**Abbreviations:** ADP: amphiphilic dendritic peptide; AgSD: silver(I) sulfadiazine; AuNC: Au nanocage; AuNR: gold nanorod; BNN6: *N*,*N*′-di-*sec*-butyl-*N*,*N*′-dinitroso-1,4-phenylenediamine; BORO: arylboronate ester; carboxy-TEMPO: nitroxide 4-carboxy-2,2,6,6-tetramethylpiperidine 1-oxyl; *α*-CD: *α*-cyclodextrin; *β*CD: *β*-cyclodextrin; Ce6: chlorin e6; COS: chitosan oligosaccharide; CPA: chitosan-*graft*-poly(amidoamine); CPCS: *N*-carboxy propionyl chitosan sodium; CS: chitosan; Cy7: cyanine7; DA: 2,3-dimethylmaleic anhydride; DEA-C3D: diethylamin-cephalosporin-3′-diazeniumdiolate; DET3: *N*-(3-(trimethoxysilyl)propyl)diethylenetriamine; EA: ethanolamine; FA: formaldehyde; GelMA: methacrylate-modified gelatin; GEN: gentamicin; GNR: gold nanorod; GNS: gold nanostar; GO: graphene oxide; GS: gentamicin sulfate; GSNO: *S*-nitrosoglutathione; HA-DA: hyaluronic acid-grafted dopamine; 3-HF: 3-hydroxyflavone; HHTP: 2,3,6,7,10,11-triphenylenehexol; HNP: hollow nanoparticle; HPDA: hollow polydopamine; IBDP: iodine-substituted dipyrromethene boron difluoride; ICG: indocyanine green; ISMN: isosorbide mononitrate; L-Arg: l-arginine; LIBDP: l-arginine-conjugated iodine-substituted dipyrromethene boron difluoride; LEVO: levofloxacin; *L*. *fermentum*: *Lactobacillus fermentum*; MET: methicillin; MIL: Material of Institute Lavoisier; MOF: metal−organic-framework; MPDA: mesoporous polydopamine; MSN: mesoporous silica nanoparticle; NOP: *N*-(3-aminopropyl)-3-(trifluoromethyl)-4-nitrobenzenamine; NP: nanoparticle; PAMAM: polyamidoamine; PAN: polyacrylonitrile; PDA: polydopamine; PDT: photodynamic therapy; PEG: poly(ethylene glycol); PEI: polyethyleneimine; PEO: poly(ethylene oxide); PET: poly(ethylene terephthalate); PFO ME: perfluorooctane microemulsion; PGMA: glycidyl methacrylate; Phe-PEU: phenylalanine-based poly(ester urea); PLGA: poly(lactic-*co*-glycolic acid); PLH: PLGA-poly-l-histidine; POEGMA: poly(oligo(ethylene glycol)methyl ether methacrylate); PROLI: proline; PTMSPA: *N*-(3-trimethoxysilyl)propyl)aniline; PTT: photothermal therapy; PVA: polyvinyl alcohol; qC: quaternized ammonium chitosan; RBNO: a boronic acid-decorated Ru^II^ compound with two NO-releasable groups; RBS: Roussin's black salt; SNAP: *S*-nitroso-*N*-acetylpenicillamine; SNO: *S*-nitrosothiol; SNP: sodium nitroprusside; TMPyP: 5,10,15,20-tetrakis(*N*-methylpyridinium-4-yl)porphyrin tetra-*p*-toluensulfonate; TSPBA: *N*^1^-(4-boronobenzyl)-*N*^3^-(4-boronophenyl)-*N*^1^,*N*^1^,*N*^3^,*N*^3^-tetramethyl-1,3-propanediaminium; UCNP: upconversion nanoparticle; ZIF-8: zeolitic imidazolate framework-8; ZnPc: zinc(II) 2,9,16,23-tetrakis(*N*-methyl-pyridiumoxy)phthalocyanine tetraiodide.

**Table 2:** CO-containing systems for antibacterial therapy.

Gas donor	System	Combined therapy or treatment	Other materials	Ref.
CORM-2	CORM-2	–	–	[[180], [181], [187], [188], [190], [191]]
CORM-2	CORM-2	Metronidazole, clarithromycin, and amoxicillin	–	[189]
CORM-2	CORM-2-conjugated polymer	–	Thiodiblock copolymer	[192]
CORM-2	P(METMA-*b*-PEGMA-CORM)		METMA-*b*-PEGMA	[193]
CORM-3	CORM-3	–	–	[[180], [181], [194], [195], [196], [197]]
[Mn(CO)_3_(tpa-κ^3^N)]Br	[Mn(CO)_3_(tpa-κ^3^N)]Br	–	–	[[198], [199], [200]]
Trypto-CORM	Trypto-CORM	–	–	[202]
Mn_2_(CO)_10_	Mn_2_(CO)_10_-embedded electrospun nonwoven	–	Poly(l-lactide-*co*-dl-lactide) nonwoven	[203]
*fac*-[Mn(CO)_3_(BZM)Br] and [RuCl_2_(BZM)(CO)_2_]	*fac*-[Mn(CO)_3_(BZM)Br] and [RuCl_2_(BZM)(CO)_2_]	–	–	[204]
3-Hydroxy-2-phenyl-4*H*-benzo[*h*]chromen-4-one	CORM-Ac	–	Acetic anhydride	[182]
CORM-401	Ce6&CO@FADP	PDT	Ce6 and FADP	[183]
Reaction between ^1^O_2_ and 3-HF	TPP-HF micelle	PDT	TPP	[205]
POS NS and the reduction of CO_2_	POS-UCNPs/ICG	PDT	UCNP and ICG	[206]
Fe_3_(CO)_12_	DNase–CO@MPDA	PTT	DNase I and MPDA	[207]

**Abbreviations:** Ce6: chlorin e6; DNase I: deoxyribonuclease I; FADP: fluorinated amphiphilic dendritic peptide; 3-HF: 3-hydroxyflavone; ICG: indocyanine green; METMA: methionine methacryloyloxyethyl ester; MPDA: mesoporous poly dopamine; PDT: photodynamic therapy; PEGMA: poly(ethylene glycol methyl ether methacrylate); POS NS: partially oxidized tin disulfide nanosheet; PTT: photothermal therapy; TPP: tetraphenylporphyrin; UCNP: upconversion nanoparticle.

### 2.1 NO donor-containing reagents, nanoparticles, hydrogels, and device

Delivering NO alone is a simple and feasible way to kill bacteria. In the past decade, various reagents, nanoparticles (NPs), hydrogels, and device capable of storing NO or loading NO donors via coordination interaction, covalent bonding, doping, or other approaches have been designed to efficiently deliver and release NO for antibacterial applications. The NO donors include diazeniumdiolate, nitroso compounds, sodium nitroprusside (SNP), isosorbide mononitrate (ISMN), furoxan, sodium nitrite (NaNO_2_), and some other synthesized NO donors. Moreover, the reaction between L-Arg and reactive oxygen species (ROS) or electrochemical NO-producing device are alternative approaches to generate NO. Through different decomposition mechanisms, these NO-releasing systems can effectively deliver NO to infection sites.

#### 2.1.1 Diazeniumdiolate

Diazeniumdiolates, also termed NONOates, are zwitterionic structures after storing 2 mol of NO (per NONOate) [67]. *N*-Diazeniumdiolates are formed via the direct reaction of gaseous NO with secondary amines in alkaline solutions. So far, *N*-diazeniumdiolates are the most widely studied NO donors due to their capacity to spontaneously release NO. As early as 2012, Jones-Carson et al. evaluated the susceptibility of *Burkholderia pseudomallei* (*B*. *pseudomallei*) to hydroxyurea, diethylamine (DETA) NONOate, and spermine NONOate, and demonstrated that *B*. *pseudomallei* bacteria are eliminated by NO in a time- and concentration-dependent fashion [74]. Later, Vumma et al. also utilized DETA NONOate to fight against uropathogenic *Escherichia coli* (*E*. *coli*) and validated the high antibacterial activity of NO released from DETA NONOate [75].

In the past years, *N*-diazeniumdiolate-containing systems have been frequently reported for NO-based antibacterial treatment, including *N*-diazeniumdiolate-modified polyamidoamine (PAMAM) conjugates [76,77], pluronic F68-branched polyethyleneimine-NONOate (termed F68-BPEI-NONOate) [78], *N*-diazeniumdiolate-containing *β*-cyclodextrin (*β*CD) derivatives [79,80], *N*-diazeniumdiolate-modified alginates [81], NONOate-loaded poly((oligoethylene glycol) methyl ether methacrylate)-*b*-poly(glycidyl methacrylate) (POEGMA-*b*-PGMA) NPs [82], PEI/NONOate-doped poly(lactic-*co*-glycolic acid) (PLGA) NPs [83] *N*-diazeniumdiolate-loaded polydopamine (PDA) hollow NPs [84], etc. As a specific example, Privett et al. synthesized diazeniumdiolate-modified proline and pressurized it to 5 atm of NO for 3 days to form PROLI/NO [85]. Then, they assessed the antibacterial activity of NO released from PROLI/NO and silver(I) sulfadiazine (AgSD) alone and in combination against five Gram-positive and four Gram-negative strains of bacteria including two antibiotic-resistant “superbugs”. It was found that Gram-negative bacteria were the most susceptible to the individual agents while Gram-positive bacteria were the most susceptible to the combination treatment. In the case of methicillin-resistant *Staphylococcus aureus* (MRSA), lower concentrations of PROLI/NO and AgSD were needed to be bactericidal in combination compared to that when used individually. In another study, Dharmaraja and coworkers synthesized arylboronate ester-based diazeniumdiolates (BORO/NO) as NO donors, which could be activated by hydrogen peroxide (H_2_O_2_) to generate NO [86]. Elevated levels of nitrite in *Pseudomonas aeruginosa* (*P*. *aeruginosa*), methicillin-sensitive *Staphylococcus aureus* (MSSA), and MRSA were observed in the presence of H_2_O_2_, leading to a high antibacterial activity of BORO/NO.

Several researchers have prepared NO-releasing chitosan derivatives in the past decade. For instance, Ji et al. crosslinked branched PEI to *N*-carboxy propionyl chitosan sodium (CPCS), followed by the reaction with NO gas in a parr high-pressure reactor to form CPCS-bPEI-NO for effective inhibition of *E*. *coli* and *Staphylococcus aureus* (*S*. *aureus*) [87]. Li and coworkers reported a PAMAM dendron-grafted chitosan (CS-PAMAM), whose secondary amine groups were reacted with NO to form CS-PAMAM/NONOate to inhibit both *E*. *coli* and *S*. *aureus* [88]. Recently, Liu et al. utilized chitosan-*graft*-poly(amidoamine) dendrimer (CPA) to yield CPA carbon dots (CPA-CDs) via a one-step hydrothermal carbonization approach [89]. Owing to the plentiful secondary amine groups on CPA, NO was subsequently loaded into CPA-CDs with the formation of *N*-diazeniumdiolate to form CPA-CDs/NONOate for the eradication of *P*. *aeruginosa*. Surprisingly, the NO loading content of CPA-CDs/NONOate was 3.5 times higher than that of CPA copolymer. *In vitro* assays showed a stronger *P*. *aeruginosa* biofilm eradication effect of CPA-CDs/NONOate than CPA-CDs. In addition, the theranostic activities of CPA-CDs and CPA-CDs/NONOate in curing *P*. *aeruginosa*-infected wounds on rats were estimated. Compared with the control group and CPA-CDs-treated group, the CPA-CDs/NONOate-treated group exhibited smaller wound areas after treatment, indicating that CPA-CDs/NONOate could accelerate wound healing process.

**Fig. 1:**
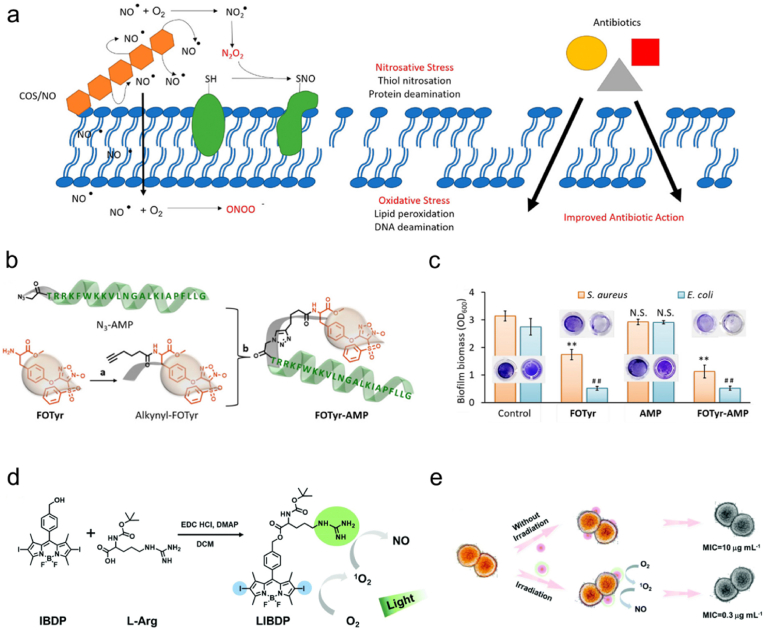
(a) Scheme depicting the antibacterial effect of COS/NO. Reproduced with permission from Ref. [92]. Copyright 2021, American Chemical Society. (b) Synthetic route of FOTyr-AMP. (c) Quantitative results of *S*. *aureus* and *E*. *coli* biofilm biomass by crystal violet staining assay. Reproduced with permission from Ref. [94]. Copyright 2020, American Chemical Society. (d) Synthetic route of LIBDP and generation processes of ^1^O_2_ and NO from LIBDP. (e) Scheme showing the antibacterial effect of LIBDP without or with light irradiation and the corresponding MIC values. Reproduced with permission from Ref. [95]. Copyright 2021, Royal Society of Chemistry.

Reighard and coworkers designed *N*-diazeniumdiolate-modified chitosan oligosaccharides as NO donor scaffolds to fight against *P*. *aeruginosa* [90,91]. Notably, an enhanced antibacterial activity was found in oxygen-free environments. The antibiofilm effectiveness of NO was better than that of tobramycin, and the bacterial phenotype showed no influence to the efficacy of this NO donor. Similarly, Rouillard et al. reported NO-releasing chitosan oligosaccharides (COS/NO) as alternatives to conventional antibiotics [92]. *N*-Diazeniumdiolate NO donors were employed in this system, and they could be decomposed into NO at physiological pH, and the generated NO could further transform into NO•, NO_2_•, ONOO^−^, and N_2_O_2_ to disturb bacterial membranes by nitrosative and oxidative stress (Fig. 1a). COS/NO not only exhibited satisfactory antibacterial efficacy in planktonic and biofilm forms, but also increased bacterial susceptibility to two traditional antibiotics tobramycin and colistin and slowed the development of antibiotic resistance. This work suggests that antibiotics in combination with NO may improve the therapeutic outcome of refractory and multidrug-resistant infections. Hall et al. synthesized *N*-diazeniumdiolate- and ethanolamine-modified chitosan oligosaccharides (COS-EA/NO), whose bactericidal action against *S*. *aureus* and *P*. *aeruginosa* was compared with that of gaseous NO under aerobic and anaerobic conditions [93]. Results proved that the COS-EA/NO required a much lower dose of NO compared with gaseous NO therapy to exert the antibacterial effect on *S*. *aureus* and *P*. *aeruginosa*, because such macromolecular NO release necessitated shorter NO diffusion distances to bacteria. Moreover, positively charged molecules, such as the chitosan scaffold, could associate with the negatively charged biofilm, further reducing the diffusion distance of NO. This work highlights the importance and necessity of developing NO delivery systems for antibacterial application.

Liu et al. reported PLGA-poly-l-histidine (PLH)-poly(ethylene glycol) (PEG) triblock charge-switchable copolymer-modified *N*-diazeniumdiolated NPs (termed PNBNPs) to eliminate *S*. *aureus* and its biofilm [96]. The PNBNPs could maintain a weak negative surface potential in physiological environment. However, when the PNBNPs reached the acidic biofilm environment, the high concentration of protons endowed the PNBNPs with a positive surface potential, thereby facilitating the release of NO. This acidity-responsive and proton-promoted NO-releasing strategy provides an option for combating acidity-associated bacterial infections and minimizing drug release in normal physiological conditions.

Kutty et al. mentioned that fimbrolides from marine algae showed potent activity against quorum sensing (QS), which is considered as a main communication and regulatory system in bacteria and controls biofilm formation and virulence factor [97]. Hence, they designed hybrid compounds consisting of nitrooxy- or diazeniumdiolate-based NO donors and fimbrolide derivatives to control biofilm development. Both biofilm inhibition assays and bioluminescent *P*. *aeruginosa* QS reporter assays revealed the antimicrobial effectiveness of the fimbrolide−NO hybrids. This work describes a dual-action antimicrobial agent with capacities of bacterial QS inhibition and NO release, and offers an alternative approach for the further development of antimicrobial agents.

#### 2.1.2 Nitroso compounds

Nitroso compounds are another kind of NO donors. Kafshgari et al. fabricated NO-, *S*-nitrosothiol (SNO)-, and *S*-nitrosoglutathione (GSNO)-loaded porous silicon NPs as bactericidal agents [98,99]. Later, Doverspike et al. mixed the as-synthesized GSNO, vaseline, and commercial zinc oxide (ZnO) cream together to obtain the NO-releasing cream GSNO/Vaseline/ZnO, wherein GSNO could naturally generate NO gas [100]. In the presence of ZnO, enhanced release of NO from GSNO was observed, and the GSNO/Vaseline/ZnO cream showed a remarkable killing effect against *Staphylococcus epidermidis* (*S*. *epidermidis*), *S*. *aureus*, and *P*. *aeruginosa*. In another study, Urzedo et al. incorporated green tea-synthesized silver NPs (AgNPs) and *S*-nitroso-mercaptosuccinic acid (an NO donor) into an alginate hydrogel for antibacterial application [101]. Owing to its viscoelastic nature, the hydrogel could be concentrated on the target area to slowly release AgNPs and NO, thus achieving effective localized antibacterial treatment.

#### 2.1.3 SNP

SNP has been a commercial NO donor for years. In 2013, Yarullina and coworkers utilized SNP to kill lactobacilli and biofilms of *Lactobacillus plantarum* (*L*. *plantarum*) [102]. When incubated with SNP, both lactobacilli and *L*. *plantarum* biofilms were remarkably inhibited. This work demonstrates the potential bactericidal ability of SNP, and later, more and more researchers choose SNP as an NO donor for antibacterial application.

#### 2.1.4 ISMN

ISMN is considered as an NO precursor and has been applied in antibacterial treatment in recent years. For example, Hasan and coworkers fabricated ISMN-loaded PLGA NPs to treat *S*. *aureus* biofilms [103]. Zhang et al. reported an ISMN immunoliposome to target and inhibit *S*. *aureus* biofilm [104]. Specifically, ISMN was doped into a liposome composed of egg lecithin and cholesterol to form an ISMN liposome. Then, the anti-*S*. *aureus* α-toxin monoclonal antibody, an *S*. *aureus* biofilm targeting molecule, was conjugated with glutaraldehyde-treated ISMN liposome to obtain ISMN immunoliposome. Results proved that the ISMN immunoliposome effectively targeted *S*. *aureus* biofilm *in vitro* and nearly completely destroyed the biofilm structure. These studies indicate that ISMN can serve as an effective NO donor in antibacterial treatments, and future research is required to determine the safety of ISMN in clinical uses.

#### 2.1.5 Furoxan

Furoxans (1,2,5-oxadiazole *N*-oxides) are another class of NO donor. Poh et al. reported three furoxan compounds (3-{[2-(dimethylamino)ethyl]oxy}-4-phenylfuroxan), 3-[(2- aminoethyl)thio]-4-phenylfuroxan, and 4-(phenylsulfonyl)- 3-{[(2-dimethylamino)ethyl]thio}furoxan) to eliminate *P*. *aeruginosa* biofilms [105]. In another study, 3-nitro-4-phenylfuroxan designed by Orlandi et al. exhibited remarkable bactericidal ability against *P*. *aeruginosa* [106]. Fei et al. reported an NO-donating antimicrobial peptide (AMP) which could treat biofilm-caused infections [94]. The as-synthesized tyrosine methyl ester-substituted furoxan derivative 4-(4-(l-alanine methyl ester-3-yl)-phenoxy)-3-(phenylsulfonyl)-1,2,5-oxadiazole-2-oxide (FOTyr) was reacted with 5-hexynoic acid to yield alkynyl-FOTyr, which was subsequently reacted with the azide derivative N_3_-AMP by a click reaction to form FOTyr-AMP (Fig. 1b). The obtained FOTyr-AMP could release NO from the furoxan head. The crystal violet staining assay indicated that FOTyr-AMP could more effectively eradicate *S*. *aureus and E*. *coli* biofilms *in vitro* compared with single FOTyr or AMP (Fig. 1c). Furthermore, an implanted biofilm infection mouse model was employed to evaluate the therapeutic efficacy of FOTyr-AMP *in vivo*. Results showed that FOTyr-AMP greatly decreased the bacterial inflammation and relieved the skin ulceration around the implanted site. This work demonstrates the excellent antibacterial and antibiofilm activities of FOTyr-AMP, and develops an effective approach for the treatment of implantable device-related biofilm infections.

#### 2.1.6 NaNO_2_

NaNO_2_ also represents a widely used NO donor. Martinez et al. utilized tetramethylorthosilicate, glucose, PEG, chitosan, and NaNO_2_ to synthesize a hydrogel/glass composite to treat MRSA wound infections [107]. The NaNO_2_ was reduced to NO through a redox reaction, and then the ingredients of the hydrogel/glass composite were dried to obtain a fine powder comprising NO-containing NPs. When exposed to an aqueous environment, the composite would open its inner water channels to facilitate the release of NO. Results showed that the hydrogel/glass composite significantly decreased minimal bacterial burden, suppurative inflammation, and collagen degradation. Zemke and coworkers reported the synergistic antimicrobial activity of NaNO_2_ and Ga(NO_3_)_3_ toward *P*. *aeruginosa* [108]. The NO produced from NaNO_2_ could cause widespread damage to Fe-containing proteins, giving rise to increased turnover of Fe–S proteins. Ga^3+^ (from Ga(NO_3_)_3_) could further replace Fe^3+^ during this increased turnover state, leading to dysfunction of metalloproteins and consequent metabolic arrest of bacteria. This work indicates that a double attack on core bacterial metabolism can be achieved by the inexpensive and stable compounds (NaNO_2_ and Ga(NO_3_)_3_), and thus this method may hold the potential for clinical use if the safety issue can be settled.

In another study, Barry et al. reported the efficacy of AB569 (a novel bactericidal tandem composed of ethylenediaminetetraacetic acid disodium salt (Na_2_-EDTA) and acidified NaNO_2_) in eliminating *P*. *aeruginosa* in murine scald burn wound and promoting wound closure and healing [95]. The antibacterial activity of AB569 was found to be attributed to the metal chelating ability of Na_2_-EDTA and the acidified NaNO_2_-mediated NO formation. Furthermore, it was also demonstrated that AB569 could reduce the proinflammatory cytokine levels and increase antiinflammatory cytokine levels, thus promoting wound healing and epidermal restoration.

In addition, Lee et al. developed an electrochemical strategy to generate NO [109]. They fabricated a small-sized polydimethylsiloxane planar patch device for antibacterial treatment. The inner chamber of the device was filled with NaNO_2_ that acted as a NO donor. With the help of a portable power, this patch could continuously generate NO over 4 days through a copper(II)-ligand catalyst-mediated electrochemical reduction of nitrite ions by an internal gold-coated steel mesh working electrode, and thus exhibited potent bactericidal effect.

#### 2.1.7 Reaction between L-Arg and ROS

The reaction between L-Arg and ROS is an effective approach to produce NO. Li et al. conjugated iodine-substituted dipyrromethene boron difluoride (IBDP) with L-Arg via a one-step esterification reaction to obtain LIBDP (Fig. 1d) to eliminate *S*. *aureus* and promote wound healing [110]. The guanidine group on LIBDP destroyed the bacterial membrane to inhibit the proliferation of *S*. *aureus*. Upon green light emitting diode (LED) light irradiation, LIBDP was proved to produce ROS, which could further oxidize the guanidine to NO to destroy the preformed biofilm. The minimum inhibitory concentration (MIC) of LIBDP was only 0.3 μg mL^−1^, which was reduced by 30 times compared with that of LIBDP without light irradiation (Fig. 1e). Moreover, LIBDP was also demonstrated to promote *S*. *aureus*-infected wound healing *in vivo*. Recently, Yu and coworkers reported an L-Arg- and H_2_O_2_-encapsulated hydrogel (L-Arg@Hydrogel/H_2_O_2_) to treat bacterial infections and promote wound healing [111]. This hydrogel was formed by mixing *N*^1^-(4-boronobenzyl)-*N*^3^-(4-boronophenyl)-*N*^1^,*N*^1^,*N*^3^,*N*^3^-tetramethyl-1,3-propanediaminium (TSPBA), polyvinyl alcohol (PVA), and L-Arg. Under the stimulation of H_2_O_2_, NO could be continuously generated from L-Arg. It was demonstrated that the generated NO could not only mediate the chemotaxis of macrophages and fibroblasts to the wound site, but also promote collagen synthesis, thereby realizing rapid wound healing and skin regeneration.

#### 2.1.8 Other NO donor or NO-producing approach

Instead of utilizing those widely used NO donors or NO-producing approaches, Tang et al. covalently immobilized 3,3-bis(aminoethyl)-1-hydroxy-2-oxo-1-triazene, a small-molecule NO donor, onto glutaraldehyde-treated chitosan to obtain chitosan-based polymeric NO, which could sustainably release NO and showed biofilm-controlling function against both Gram-negative and Gram-positive bacteria [112].

Owing to the high electronegativity of fluorine, perfluorooctane (PFO) possesses excellent NO affinity. Choi et al. utilized such property of PFO to load NO for the elimination of *S*. *aureus* [113]. They developed a nanoscale pluronic F-127 microemulsion (ME), in which PFO was loaded via ultrasonication for just 10 min. The as-prepared PFO ME was treated with an NO gas stream for 2 h to incorporate NO and thus it could continuously release NO for 12 h. Results showed that PFO ME remarkably accelerated the death of *S*. *aureus*. This work provides a simple and time-saving method to fabricate antibacterial agents.

### 2.2 NO-containing coatings, surfaces, or films

Surface decoration is usually utilized to prevent bacterial adhesion or biofouling. Attaching NO donors to different coatings, surfaces, or films exhibits great potential for antibacterial applications. For instance, Storm et al. sprayed a fluorinated silane/silica composite on *N*-diazeniumdiolate-modified xerogel and thus obtained a superhydrophobic NO-releasing xerogel to reduce bacterial adhesion and kill adhered bacteria on the surface of the xerogel [114]. In another study, Sadrearhami et al. functionalized a glass substrate with PEG-grafted PDA, followed by purging with NO gas to form *N*-diazeniumdiolate moieties (NO precursors) [115]. This NO-releasing PDA coating exhibited 97%, 99.9%, and 99% killing activities against surface-attached *P*. *aeruginosa* PA37, *P*. *aeruginosa* PAO1, and *S*. *aureus*, respectively. Recently, Jeong et al. fabricated multilayered nanofilm (nbi film) by alternative deposition of branched PEI and alginate [116]. Then, *N*-diazeniumdiolate was formed at the secondary amine moiety of branched PEI and Cu(II) ion was incorporated by forming chelating compounds to obtain nbi/NO/Cu film for eradication of *S*. *aureus* and *P*. *aeruginosa*.

*S*-Nitroso compounds also serve as NO donors for surface functionalization. Kim et al. developed GSNO-loaded chitosan films to fight against *P*. *aeruginosa* and *S*. *aureus* [117]. Sundaram et al. incorporated *S*-nitroso-*N*-acetylpenicillamine (SNAP, another kind of NO donor) into nanocellulose−chitosan layer to fabricate biodegradable antimicrobial composite membranes for the inhibition of *Enterococcus faecalis* (*E*. *faecalis*), *Listeria monocytogenes*, and *S*. *aureus* [118]. After that, Sadrearhami and coworkers fabricated SNO-functionalized coatings via plasma polymerization of 3-mercapto-3-methylbutan-1-ol monomer and subsequent nitrosation with *tert*-butyl nitrite to fight against *P*. *aeruginosa* [119]. Additionally, several studies also reported that SNAP could be immobilized within CarboSil 20 80A, a thermoplastic silicone-polycarbonate-urethane biomedical polymer, to reduce bacterial attachment or prevent biofilm formation [[120], [121], [122]].

NO-releasing coatings have been applied in indwelling medical devices as well. Some researchers impregnated the commercial silicone Foley catheter with SNAP via a solvent swelling method [123,124]. One of the SNAP-incorporated silicone Foley catheters prepared by Colletta et al. could generate NO under physiological conditions for over one month, and significantly decreased formation of biofilm on its surface over a 14-day period [123]. Mihu and coworkers applied the hydrogel/glass composite in a rat central venous catheter model to prevent the adhesion and formation of biofilms *in vivo* [125]. Fleming et al. immobilized two kinds of aminosilane molecules, *N*-(3-trimethoxysilyl)propyl)aniline (PTMSPA) and *N*-(3-(trimethoxysilyl)propyl)diethylenetriamine (DET3), on silicone elastomer (SE) and poly(ethylene terephthalate) (PET) (two polymers widely used as coatings of indwelling medical devices) to form *N*-diazeniumdiolates as NO donors [126]. The obtained NO-releasing coatings significantly reduced the adhesion of *P*. *aeruginosa* over 24 h. Recently, Li and coworkers decorated 6-aminohexyl-3-aminopropyltrimethoxysilane and 11-aminoundecyltriethoxysilane on Ti rods to prevent adhesion of *S*. *aureus* and *P*. *aeruginosa* in orthopedic applications [127]. The formation of diazeniumdiolates on the Ti surface effectively inhibited *S*. *aureus* and *P*. *aeruginosa* in 24 h, and the diazeniumdiolate-functionalized Ti rods also showed cytocompatibility toward primary human osteoblast cells. These two examples illustrate the potential and feasibility of the NO donor coating strategies in inhibiting bacterial growth and biofilm formation on the surfaces of indwelling medical devices.

Several studies used electrospun films as NO carriers for antibacterial applications. For example, Worley et al. prepared octyl alkyl chain- or quaternary ammonium (QA) moiety-functionalized PAMAM dendrimers, whose secondary amines were subsequently modified with *N*-diazeniumdiolates [128]. Then, the resulting dendrimers were added into polyurethane solutions, followed by electrospinning to form dendrimer-doped composite polyurethane fibers as an antibacterial wound dressing. Dolansky et al. fabricated an *N*-(3-aminopropyl)-3-(trifluoromethyl)-4-nitrobenzenamine (NOP, an NO donor)- and PEI-bonded electrospun polystyrene (PS) nanofiber membrane (PS-PEI/NOP) to kill *E*. *coli* [129]. In another study, Workman and coworkers prepared SNAP-PAN fibers through covalently attaching SNAP to PAN fibers [130]. Owing to the sustained release of NO from SNAP, the SNAP-PAN fibers not only exhibited a 99.71% reduction in the number of adhered *S*. *aureus* compared with PAN fibers, but also affected the bacterial growth surrounding the fibers. Moreover, the SNAP-PAN fibers could accelerate the wound healing process by capturing and removing exudates from the pores of the fibers. Surprisingly, it was also found that the SNAP-PAN fibers increased the proliferation and attachment of fibroblasts around the wound site. Recently, Li et al. fabricated an electrospun composite film to eradicate *S*. *aureus* and promote *S*. *aureus*-infected wound repair [131]. This film was obtained by grafting phenylalanine-based poly(ester urea)s (Phe-PEUs) to polyacrylonitrile (PAN) via electrospinning, followed by the modification with GSNO to obtain Phe-PEUs/PAN-G with the ability to release NO. The resultant film possessed high thermal stability and was able to stably and continuously release NO for 360 h. After applied to the *S*. *aureus*-infected wounds on mice, Phe-PEUs/PAN-G could release NO from GSNO to kill *S*. *aureus* on the surface of the wounds. Subsequently, NO could further permeate into biofilms to exert its antibiofilm activity. Moreover, it was proved that Phe-PEUs/PAN-G could increase the synthesis of collagen and promote the proliferation of fibroblasts around the wound areas, thus accelerating wound healing. The above two studies successfully construct multifunctional NO-releasing wound dressings that combine both antibacterial and wound healing promotion functions.

### 2.3 Light-triggered NO-releasing systems

Accurate delivery and controlled release of NO in infected tissues are challenging but significant. Dong et al. impregnated the mesoporous upconversion nanoparticle (UCNP) NaGdF_4_:Yb/Tm@mSiO_2_ (UCNP@mSiO_2_, mSiO_2_ stands for mesoporous silica) with Roussin's black salt (RBS, a kind of NO donor molecule), and the as-formed product was subsequently coated with quaternized ammonium chitosan (qC) to construct a near-infrared (NIR)-triggerable NO delivery nanocompound (termed RBS@UCNP@mSiO_2_@qC) for synergistic elimination of the antibiotic-resistant bacteria-based biofilms [132]. The UCNPs could harvest NIR light and transfer the energy to the light with a specific wavelength, which could lead to the NO release from RBS. The cationic polymer qC was used to combat drug-resistant bacteria because it could disrupt the cytoplasmic membrane of the bacteria. The authors revealed that RBS@UCNP@mSiO_2_@qC showed a remarkable synergistic eradication effect on the antibiotic-resistant bacteria-based biofilms *in vitro*. Additionally, they demonstrated that the nanocompound had negligible toxicity to mammalian cells. With its synergistic antibacterial effect and satisfactory biocompatibility, this multifunctional NIR-responsive NO delivery nanoplatform provides a new way to combat antibiotic-resistant biofilm-associated infections. Yu et al. imbedded ruthenium nitrosyl (Ru–NO)-functionalized Fe_3_O_4_@PDA into chitosan–PVA to develop a thermosensitive CS-PVA/NO hydrogel [133]. Under mild 808 nm light illumination, the CS-PVA/NO hydrogel could steadily release NO to kill both *S*. *aureus* and *E*. *coli*.

**Fig. 2:**
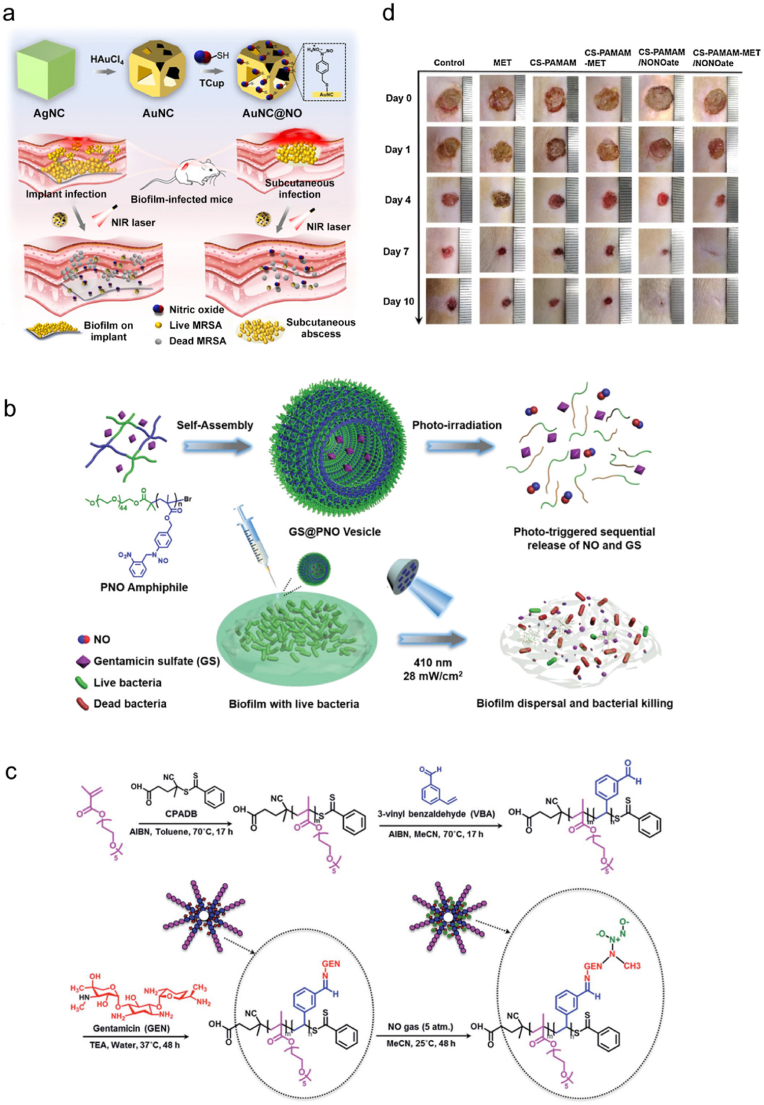
(a) Synthetic route of AuNC@NO and its antibacterial effect on MRSA biofilm-infected mice. Reproduced with permission from Ref. [134]. Copyright 2021, American Chemical Society. (b) Scheme illustrating the formation of GS@PNO vesicle and its antibacterial effect on *P*. *aeruginosa* biofilm upon 410 nm light irradiation. Reproduced with permission from Ref. [135]. Copyright 2021, Wiley-VCH. (c) Synthetic route of POEGMA-*b*-PVBA-GEN. Reproduced with permission from Ref. [136]. Copyright 2016, Royal Society of Chemistry. (d) Photographs showing the healing process of wounds treated with MET, CS-PAMAM, CS-PAMAM-MET, CS-PAMAM/NONOate, or CS-PAMAM-MET/NONOate at different days during treatments. Reproduced with permission from Ref. [137]. Copyright 2020, Elsevier.

Tang et al. developed NIR-stimulated NO-releasing Au nanocages to kill MRSA and its biofilm [134]. Specifically, they first fabricated Au nanocages (AuNCs) via a galvanic replacement reaction of the as-prepared Ag nanocubes (AgNCs) and HAuCl_4_, and then thiolate cupferron (TCup, with a full name of *N*-nitroso(4-mercaptomethylphenyl)-hydroxylamine, a temperature-sensitive NO donor) was loaded on the surface of the AuNCs to form AuNC@NO (Fig. 2a). AuNC@NO was demonstrated to slowly and continuously release NO from TCup at a physiological temperature or realize quick NO release under 808 nm NIR light irradiation. Compared with AuNCs, AuNC@NO exhibited enhanced bactericidal and antibiofilm efficacies *in vitro*. Additionally, a subcutaneous MRSA biofilm infection model and an implant MRSA biofilm infection model were separately established to estimate the antiinfective capacity of AuNC@NO. Upon NIR light irradiation, AuNCs were quickly heated and a large amount of NO was released, achieving a remarkable inhibition of MRSA biofilm. Duan and coworkers fabricated an amphiphilic diblock copolymer poly(ethylene oxide)-*b*-poly(4-((2-nitrobenzyl)(nitroso)amino)benzyl methacrylate) (PEO-*b*-PNBM, abbreviated as PNO) (Fig. 2b) [135]. The resultant PNO could self-assemble into vesicles, which could encapsulate gentamicin sulfate (GS) into their hydrophilic lumens to obtain GS@PNO vesicles to eradicate *P*. *aeruginosa* PAO1 biofilms. To be noted, the 410 nm light irradiation could trigger cleavage of N–NO bonds and sequentially release NO and GS, thereby realizing biofilm dispersal and bacterial eradication.

In another study, Wu et al. fabricated a nanogenerator SNP@MOF@Au-Mal for the treatment of *P*. *aeruginosa*-infected wounds [138]. In this system, SNP was loaded into the MOF (MIL-101-NH_2_; MIL stands for Material of Institute Lavoisier), on whose surface a layer of gold shell was grown in situ to endow it with NIR light absorption property. Finally, carboxyl-PEG_5000_-maleimide (HOOC-PEG_5000_-Mal) was wrapped on the gold shell to form SNP@MOF@Au-Mal. Owing to the exposed maleimide, this nanogenerator could specifically recognize and attach to the type IV pilus (T4P) of *P*. *aeruginosa* and then release NO and generate ROS upon NIR light irradiation to realize synergistic antibacterial effect. *In vivo* experiments demonstrated that the bacterial burden in the wound was reduced by 97.7%. Moreover, it was found that SNP@MOF@Au-Mal could shorten the M1 polarization cycle and induce the premature polarization of the M1 macrophages to M2 macrophages in infected skin tissues, thus promoting the secretion of growth factors for wound healing. Collectively, this work not only demonstrates the potent bactericidal effect and potential wound healing ability of SNP@MOF@Au-Mal, but also proposes an effective and promising approach for precise treatment of *P*. *aeruginosa* infections. Yang et al. also chose SNP as an NO donor and developed an NIR-triggered NO nanogenerator to kill bacteria in infectious diabetic ulcers and promote wound healing [139]. The authors first prepared a UCNP as a core, on which zeolitic imidazolate framework-8 (ZIF-8) grew as a MOF shell, and then loaded SNP into the ZIF-8 layer to obtain SNP@MOF-UCNP. Next, ssPDA (an ROS-responsive compound with a disulfide bond) was coated onto the MOF shell, and cyanine7 (Cy7) and IR786s were adsorbed on the ssPDA surface by π–π interactions to form the final product SNP@MOF-UCNP@ssPDA-Cy7/IR786s (abbreviated as SNP@UCM). Usually, a large amount of ROS exist in infected tissues because of the mitochondrial injury. As a result, the ROS could react with NO released from SNP@UCM under 980 nm NIR irradiation to form ONOO^−^, thus disrupting bacterial membrane integrity and realizing pathogen clearance. Moreover, SNP@UCM also possessed the diagnosis ability of bacterial infection. The fluorescence of Cy7 was quenched by IR786s in bacteria-free conditions. However, during infection, a sharp increase in ROS could lead to the decomposition of ssPDA and separation of Cy7 and IR786s, giving rise to a strong fluorescence signal. Additionally, *in vivo* assays demonstrated that SNP@UCM could simultaneously eliminate bacteria, accelerate wound healing, reduce inflammation, and promote angiogenesis in infected diabetic ulcers.

Very recently, Liang and coworkers prepared a gold nanostar/hollow PDA (GNS/HPDA) Janus nanostructure via a modified seed-mediated synthetic method, and then loaded *N*,*N*′-di-*sec*-butyl-*N*,*N*′-dinitroso-1,4-phenylenediamine (BNN6, an NO donor) onto the GNS/HPDA Janus nanostructure through π–π stacking interaction to form GNS/HPDA-BNN6 [140]. Upon 808 nm light irradiation, this system realized precise NIR light-controlled NO release to kill MRSA. Chen et al. reported a catalysis-triggered NO-releasing system [141]. Specifically, they encapsulated the photocatalyst *fac*-Ir(ppy)_3_ into the *N*,*N*′-dinitroso-1,4-phenylenediamine-based triblock copolymer-derived micelle (forming Ir@PBNN-NO_2_) to treat MRSA infections *in vivo*. The photocatalyst *fac*-Ir(ppy)_3_ could selectively activate *N*,*N*′-dinitroso-1,4-phenylenediamine derivatives under 500 nm light irradiation to form quinonediimine (QDI) residues and release NO. The QDI derivatives could scavenge the in situ generated ROS and thereby facilitate self-promoted oxygen depletion. This system realizes photoredox catalysis-triggered NO release and oxygen scavenging for efficient treatment of MRSA infections, demonstrating the potential of photoredox catalysis in antibacterial applications.

### 2.4 Combination of NO therapy and antibiotic treatment

Combination therapy represents a robust strategy to kill the drug-resistant bacteria [142]. The concerted use of NO and other antibiotics is found to be more effective than the NO and antibiotics administered individually with equivalent doses, thus making the combination of NO and antibiotics a superior choice to reduce dose-related toxicity to normal tissues and cost of treatment. For instance, Bi et al. synthesized ocotillol-type nitrate derivatives and combined them with chloramphenicol and kanamycin for synergistic elimination of *S*. *aureus*, *Bacillus subtilis*, and *E*. *coli* [143]. Reffuveille et al. reported that the combined use of nitroxide 4-carboxy-2,2,6,6-tetramethylpiperidine 1-oxyl (termed carboxy-TEMPO) and ciprofloxacin exhibited synergistic treatment effect toward the biofilms formed by Gram-negative bacteria [144]. This therapy not only dispersed mature biofilms but also enhanced the eradication activity of ciprofloxacin. Results showed that the biofilms formed by *P*. *aeruginosa* and *E*. *coli* were reduced by 99.3% and 93%, respectively. Craven and coworkers used NO-releasing PAN (termed PAN/NO) to inhibit a multispecies biofilm composed of *S*. *aureus*, *P*. *aeruginosa*, and *E*. *faecalis* [145]. PAN/NO dispersed the multispecies biofilm and remarkably reduced the viability of the biofilm with the help of ciprofloxacin. Later, Yang and coworkers prepared hyperbranched polyaminoglycosides by the polymerization of *N*,*N*′-methylenebis(acrylamide), kanamycin, gentamicin (GEN), and neomycin via a one-pot reaction to kill common dental pathogens [146]. Hasan et al. combined an ISMN-loaded chitosan gel (CS-ISMN) with ciprofloxacin to kill *S*. *aureus* bioflim, and a strong synergy of CS-ISMN and ciprofloxacin was observed [147].

In another study, Nguyen and coworkers fabricated NO-loaded block copolymer POEGMA-*b*-PVBA-GEN to fight against the biofilm of *P*. *aeruginosa* [136]. Specifically, POEGMA was first synthesized by the reaction between 4-cyanopentanoic acid dithiobenzoate (CPADB) and oligo(ethylene glycol) methacrylate (OEGMA) in toluene at 70 °C for 17 h in the presence of 2,2′-azobisisobutyronitrile (AIBN) (Fig. 2c). Then, POEGMA was chain extended in the presence of 3-vinylbenzylaldehyde (VBA) and AIBN in methyl cyanide (MeCN) at 70 °C for 17 h to form POEGMA-*b*-PVBA, which was subsequently reacted with GEN in water in the presence of triethylamine (TEA) at 37 °C for 48 h to afford POEGMA-*b*-PVBA-GEN. Finally, it was exposed to 5 atm of NO in MeCN at 25 °C for 48 h for NO loading. The authors demonstrated that the NO-loaded POEGMA-*b*-PVBA-GEN complex realized a sustainable and simultaneous release of GEN and NO to eliminate the biofilms formed by *P*. *aeruginosa*. The complex was found to remarkably disperse the biofilms and strongly decrease the viability of planktonic cells and biofilms by over 95% and 90%, respectively, while using GEN or NO alone only decreased the biofilm viability by less than 20%.

Kelso's group designed a cephalosporin-3′-diazeniumdiolate as an NO-donor prodrug, which can selectively target bacterial infection sites and be activated by a bacteria-specific enzyme β-lactamase to realize NO release [[148], [149], [150]]. Based on this, Collins et al. reported that cephalosporin-3′-diazeniumdiolate treatment remarkably increased the susceptibility of nontypeable *Haemophilus influenzae* (NTHi) biofilms to azithromycin [151]. Later, Soren et al. fabricated a diethylamin-cephalosporin-3′-diazeniumdiolate (DEA-C3D) NO-donor prodrug, which was combined with two traditional antibiotics, tobramycin and colistin, to eradicate *P*. *aeruginosa* biofilms [152]. Specifically, the biomass of *P*. *aeruginosa* biofilm was reduced by 50.9% with DEA-C3D treatment, by 89.8% with colistin treatment, and by 97.8% with the combined treatment of DEA-C3D and colistin. Similarly, DEA-C3D in combination with tobramycin also exhibited remarkable reduction in biofilms *in vitro* relative to the use of DEA-C3D alone.

Recently, Liu et al. conjugated *N*-diazeniumdiolates and antibiotic methicillin (MET) to chitosan-*graft*-poly(amidoamine) dendrimer to form CS-PAMAM-MET/NONOate [137]. This codelivery system exhibited combined and effective antibacterial activity to *S*. *aureus* and *E*. *coli in vitro*. Additionally, compared with MET, CS-PAMAM, CS-PAMAM-MET, and CS-PAMAM/NONOate, CS-PAMAM-MET/NONOate remarkably accelerated the healing of MRSA-infected wounds *in vivo* after 10 days of treatment (Fig. 2d). With their ability to thoroughly eradicate a wide range of bacteria, the combination of NO and MET can be particularly useful in treating polymicrobial and antibiotic-resistant infections and reduce the doses of antibiotics at the same time, which ensures the great potential of these combination therapies in clinical applications. da Silva Filho et al. loaded SNP in silica NP (MPSi) to form an NO-releasing system MPSi-NP, which exhibited remarkable biofilm inhibition effect toward MSSA and MRSA [153]. Furthermore, the authors integrated MPSi-NP with a traditional antibiotic ampicillin or tetracycline, and realized significantly enhanced bactericidal effect toward MRSA.

Ren and coworkers proposed an electrochemical NO-releasing catheter device for eradication of mature *P*. *aeruginosa* biofilms [154]. Specifically, copper(II)-tri(2-pyridylmethyl)amine, NaCl, NaNO_2_, and 4-(2-hydroxyerhyl)piperazine-1-erhanesulfonic acid (HEPES) buffer were filled into a single lumen silicone catheter, and an Ag/AgCl wire and a Teflon-coated Pt wire were inserted into the lumen as reference/counter and working electrodes, respectively. The controllable release of NO could be realized by applying a certain voltage. Moreover, NO greatly enhanced the antibacterial efficacies of colistin, GEN, chloramphenicol, ciprofloxacin, tetracycline, and beta-defensin 2. This work presents the precise control of NO release by an electrochemical method, and also demonstrates the synergistic effect between NO and other antibacterial agents.

### 2.5 Combination of NO therapy and phototherapy (PTT/PDT)

**Fig. 3:**
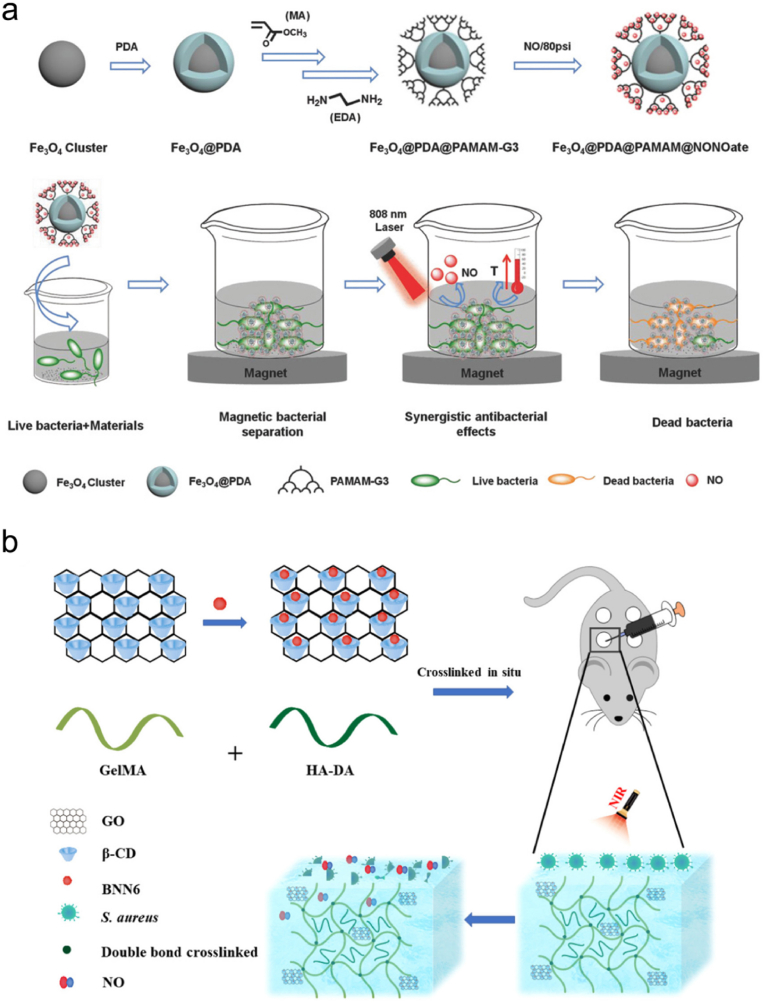
(a) Synthetic route of Fe_3_O_4_@PDA@PAMAM@NONOate and its application in magnetic separation and synergistic NO and photothermal killing of bacteria. Reproduced with permission from Ref. [155]. Copyright 2018, Wiley-VCH. (b) Schematic illustration of the synthetic route and antibacterial effect of GO-*β*CD-BNN6. Reproduced with permission from Ref. [157]. Copyright 2020, American Chemical Society.

Antibacterial PTT has attracted intensive attention due to its high specificity and capacity to induce bacterial apoptosis. For example, Yu and coworkers reported a multifunctional NO-releasing and photothermal nanocomposite to eliminate *E*. *coli* and *S*. *aureus* [155]. They first developed a PDA-coated iron oxide nanocomposite (Fe_3_O_4_@PDA) as a core (Fig. 3a). After reacting with methyl methacrylate (MA) and ethylenediamine (EDA), Fe_3_O_4_@PDA was grafted by three generation dendritic poly(amidoamine) (PAMAM-G3), and NO was loaded at 80 psi via the formation of NONOate. The resultant Fe_3_O_4_@PDA@PAMAM@NONOate could lead to a sharply increased temperature and controllable NO release under intermittent 808 nm laser irradiation, thereby achieving a synergistic bactericidal effect. Moreover, due to the existence of Fe_3_O_4_, the bacteria and the nanocomposite could be magnetically removed from the corresponding environments within 10 min. Garcia et al. developed a nanoassembly with photothermal and antimicrobial capacities to fight against *S*. *aureus* biofilm [156]. In this system, a gold nanorod (AuNR) was first fabricated as a core, and a mesoporous silica NP (MSN) shell was coated on it to form a core–shell structure termed AuNR@MSN. Then, the AuNR@MSN was functionalized with nitrosothiol group that acted as an NO donor, and levofloxacin (LEVO) was loaded into AuNR@MSN as an antibiotic. Under 808 nm light irradiation, the temperature rise of this nanoassembly could cause the photothermal effect and stimulate the release of NO and LEVO, giving rise to a *S*. *aureus* biofilm reduction of 90%.

Huang et al. loaded graphene oxide (GO), *β*CD, and BNN6 into a methacrylate-modified gelatin (GelMA)/hyaluronic acid-grafted dopamine (HA-DA) hydrogel to form the adhesive Gel/GO-*β*CD-BNN6 nanocomposite hydrogel for the bacteria-infected wound repair (Fig. 3b) [157]. The combination of the photothermal effect of this hydrogel and NO released from BNN6 under NIR light irradiation improved the antibacterial performance both *in vitro* and *in vivo*. Besides, this hydrogel also enhanced angiogenesis and collagen deposition to facilitate wound healing. This work presents a multifunctional nanocomposite hydrogel with antibacterial and angiogenic properties, greatly promoting the healing of bacteria-infected wounds.

However, utilizing PTT alone may be ineffective to heat-resistant bacteria [158]. Fortunately, synergistic PDT and PTT can well overcome the PTT treatment ineffectiveness issue of heat-resistant bacterial species. For example, Yuan et al. loaded indocyanine green (ICG) into L-Arg-modified mesoporous polydopamine (MPDA) to obtain a phototherapeutic nanoplatform (termed AI-MPDA) to eliminate the already-formed biofilm [159]. Under NIR light irradiation, ICG was first released and stimulated to produce ROS. Subsequently, L-Arg produced NO in the presence of ROS, and NO further reacted with ^1^O_2_ to generate reactive (ONOO^−^) for NO-enhanced PDT. Very recently, Qi and coworkers loaded SNO and ICG into mSiO_2_-coated gold nanorods (GNRs) to form NIR light-triggerable NPs (termed GNRs@mSiO_2_-SNO/ICG) with antibacterial and antiinflammatory abilities to treat periodontal disease [160]. Upon a single 808 nm light irradiation, the synergistic antibacterial effect could be realized not only by NO generated from SNO, but also by PTT-triggered biofilm dispersal and PDT-induced bacterial killing.

**Fig. 4:**
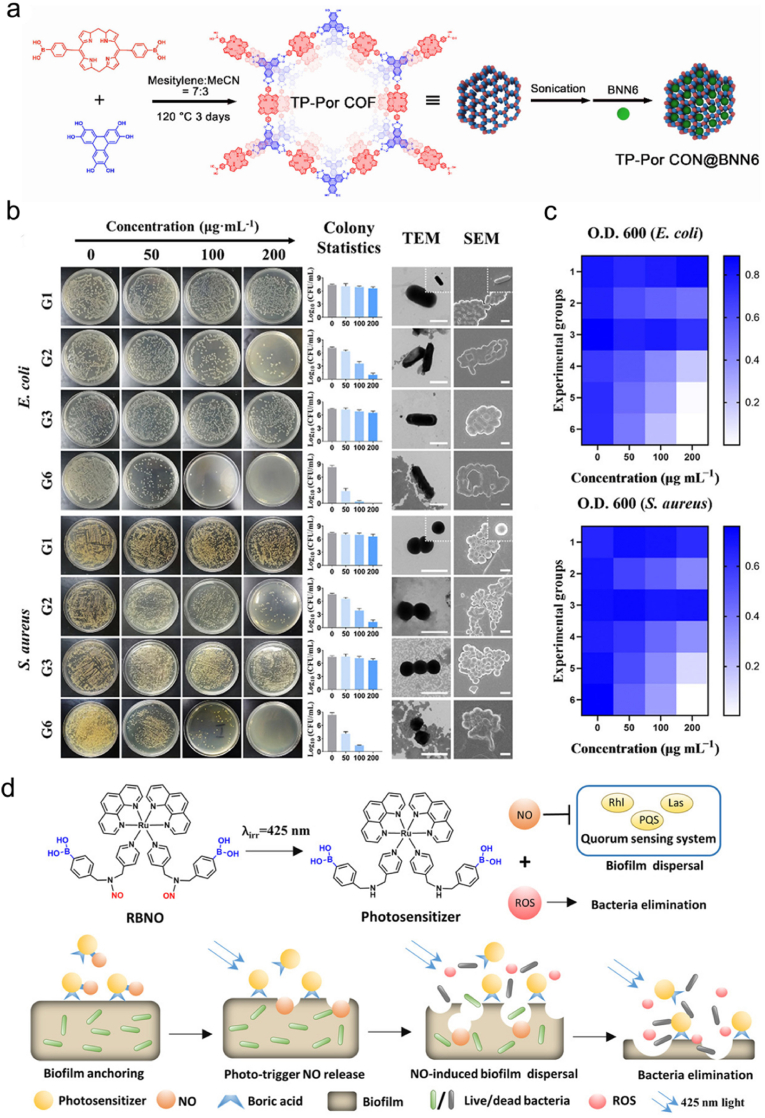
(a) Schematic illustration of the synthetic route of TP-Por CON@BNN6. (b) Colony plate photographs, colony statistics, and representative TEM and SEM images of *E*. *coli* and *S*. *aureus* after different treatments. Group 1 (G1): TP-Por CON, group 2 (G2): TP-Por CON + PDT + PTT, group 3 (G3): TP-Por CON@BNN6, group 4 (G4): TP-Por CON@BNN6 + PDT, group 5 (G5): TP-Por CON@BNN6 + PDT + PTT, group 6 (G6): TP-Por CON@BNN6 + PDT + PTT + GT (gas therapy). (c) Checkerboard of the O.D. 600 values of *E*. *coli* and *S*. *aureus* after treatment with different groups at different concentrations. Reproduced with permission from Ref. [161]. Copyright 2021, American Chemical Society. (d) Scheme showing the capacity of RBNO to generate NO and ROS upon 425 nm irradiation to eliminate *P*. *aeruginosa* biofilm. Reproduced with permission from Ref. [162]. Copyright 2021, Wiley-VCH.

In another study, Sun et al. synthesized porphyrin-based porous covalent organic framework (COF) nanosheets (CON) via an esterification reaction between 2,3,6,7,10,11-triphenylenehexol (HHTP) and 5,15-bis(4-boronophenyl)-porphyrin, and then BNN6 was encapsulated into the pores of the nanosheets to obtain the final product TP-Por CON@BNN6 (Fig. 4a) for killing *E*. *coli* and *S*. *aureus* bacteria [161]. In this system, TP-Por CON showed remarkable PDT and PTT efficacies under single 635 nm light irradiation, while BNN6 could simultaneously release NO to realize gas therapy. As shown in Fig. 4b, different groups were set to evaluate the bactericidal activity of TP-Por CON and TP-Por CON@BNN6. Both *E*. *coli* and *S*. *aureus* treated with group 6 (G6) showed the highest mortality rate. Meanwhile, the transmission electron microscopy (TEM) and scanning electron microscopy (SEM) images revealed that the G6-treated bacteria became fractured and wrinkled compared with other groups. The optical densities at 600 nm (O.D. 600) of the bacteria treated with different groups at different concentrations were also summarized on the checkerboard (Fig. 4c, the lighter blue indicates a lower bacterial density). Consistent with the above results, a significant bacterial clearance efficacy was observed in G6 at 200 μg mL^−1^. Collectively, this work combines COF-based material with NO donor and integrates the effects of gas therapy, PDT, and PTT using a single irradiation source, and highlights the advantages and potential of such synergistic therapy in antibacterial applications.

**Fig. 5:**
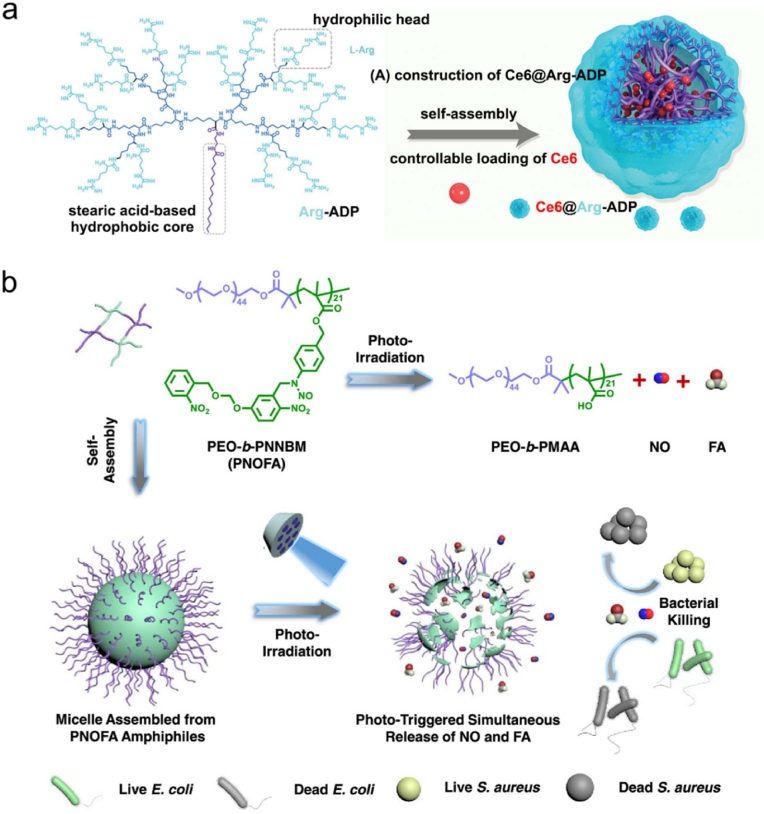
(a) Scheme showing the fabrication route of Ce6@Arg-ADP. Reproduced with permission from Ref. [165]. Copyright 2021, Wiley-VCH. (b) Scheme illustrating the self-assembly process of PEO-*b*-PNNBM (PNOFA) to PNOFA micelle and the photoirradiation-mediated conversion of PNOFA to PEO-*b*-PMAA, NO, and FA to kill both *E*. *coli* and *S*. *aureus*. Reproduced with permission from Ref. [166]. Copyright 2021, American Chemical Society.

Regarding the combination of PDT and NO therapy, Dolansky et al. covalently grafted NOP and photosensitizer (5,10,15,20-tetrakis(*N*-methylpyridinium-4-yl)porphyrin tetra-*p*-toluensulfonate (TMPyP) or zinc(II) 2,9,16,23-tetrakis(*N*-methyl-pyridiumoxy)phthalocyanine tetraiodide (ZnPc)) into electrospun PS nanofibers to form NOP/TMPyP or NOP/ZnPc, thereby realizing the synergistic bactericidal activity against *E*. *coli* [163,164]. In another study, Zhao et al. synthesized a boronic acid-decorated Ru^II^ compound with an NO-releasable group (termed RBNO) as both a light-triggered NO-releasing agent and a boronic acid-decorated photosensitizer to eradicate *P*. *aeruginosa* biofilms [162]. As shown in Fig. 4d, NO could be released and ROS could be produced simultaneously upon 425 nm light irradiation. Notably, several essential proteins in the QS system were identified to be *S*-nitrosylated by NO, thus giving rise to biofilm dispersal. Moreover, the boronic acid enabled the RBNO to selectively anchor to the extracellular polysaccharides of *P*. *aeruginosa*, thereby endowing RBNO with a targeting capability toward *P*. *aeruginosa* biofilms. Zhu et al. fabricated an L-Arg-rich amphiphilic dendritic peptide (Arg-ADP) as a carrier, followed by a self-assembly process of chlorin e6 (Ce6) in aqueous solution to obtain a PDT-driven NO controllable delivery system Ce6@Arg-ADP (Fig. 5a) for the treatment of subcutaneous abscesses [165]. It was found that Ce6@Arg-ADP exhibited superb abilities to associate with bacteria and penetrate biofilms. After efficient penetration into biofilms, Ce6@Arg-ADP could rapidly produce massive ^1^O_2_ and H_2_O_2_ during the 665 nm light-triggered PDT process, and the generated H_2_O_2_ could oxidize Arg-ADP to produce NO. The authors substituted l-lysine (Lys) for Arg and set the resultant Ce6@Lys-ADP as a control group, and it was found that upon 665 nm light irradiation, the synergistic biofilm eradication effect of Ce6@Arg-ADP was better than that of Ce6@Lys-ADP, confirming that Arg-ADP could be oxidized to NO for antibacterial effect. Moreover, following efficient elimination of bacteria at the abscess site, trace quantities of NO could be further generated to facilitate the angiogenesis and epithelialization of the wound tissues, thus promoting the wound healing *in vivo*. This study provides a PDT-driven NO controllable generation strategy with remarkable antibacterial and antibiofilm efficacies, and highlights that the combination of PDT and NO therapy represents a promising way to treat subcutaneous abscesses or other biofilm-caused infections.

Hu and coworkers proposed a surface charge-switchable supramolecular nanocarrier for eradicating MRSA biofilms via NO-facilitated PDT [167]. In this system, the PEG block polypeptide copolymer (PEG-(KLAKLAK)_2_) was modified with 2,3-dimethylmaleic anhydride (DA) to form a PEG-(KLAKLAK)_2_-DA that exhibited pH-sensitive charge reversal property, which subsequently interacted with glutathione (GSH)-responsive *α*-cyclodextrin (*α*-CD)-based prodrugs (Ce6-decorated *α*-CD and SNAP-decorated *α*-CD) to obtain the supramolecular nanocarrier *α*-CD-Ce6-NO-DA. At physiological pH (7.4), the *α*-CD-Ce6-NO-DA nanocarrier exhibited negatively charged surface, while it realized completed charge reversal and became positively charged at the acidic biofilm pH (5.5), thus facilitating adhesion to negatively charged bacterial surface. After penetration into biofilm, *α*-CD-Ce6-NO-DA could be triggered by the overexpressed GSH to release NO. Ce6 exerted PDT effect upon 660 nm laser irradiation, and the depletion of GSH prevented the reaction between ROS and GSH during PDT, thereby enhancing the PDT efficacy.

### 2.6 Bacteria-mediated NO generation

*Lactobacillus fermentum* (*L*. *fermentum*) is a kind of lactic acid-producing bacteria, which can react with glucose to generate lactic acid and the protons of lactic acid molecules will further react with nitrite to produce NO [168]. Jones et al. developed an NO-producing probiotic adhesive patch composed of *L*. *fermentum*, nitrite salt (NaNO_2_), and glucose [168]. This NO-producing probiotic patch could cause complete death of *S*. *aureus*, *E*. *coli*, *P*. *aeruginosa*, MRSA, *Trichophyton rubrum*, and *Trichophyton mentagrophytes* in 4–8 h. This work demonstrates the capacity of NO to eradicate a wide spectrum of bacteria and introduces NO-producing patches to antibacterial treatment.

After that, Hibbard et al. synthesized nitroaromatic-protected piperazine diazeniumdiolate prodrugs to kill *E*. *coli* [169]. The nitroreductase, an enzyme almost exclusively exists in bacteria, can reduce the nitroaromatic-protecting group and catalyze NO release to kill bacteria. The antibacterial activity of the compound was evaluated, and a 94% reduction in the number of *E*. *coli* was found at a concentration of 1 mM. When *E*. *coli* was exposed to the synthesized diazeniumdiolates, significant NO release and reduction in the number of bacteria were observed, while no NO was released in the absence of this enzyme. This work validates the possibility of realizing site-specific delivery of antimicrobial agents to infections, which can not only improve the antimicrobial efficacy but also reduce the side effects of antibiotics to normal tissues.

### 2.7 Synergistic gas therapy using NO and other gases

Gao et al. reported a system which can simultaneously release CO and NO from a single donor molecule upon 410 nm light irradiation to kill *S*. *aureus* and treat MRSA infections [170]. Specifically, the NO-releasing *N*-nitrosamine moiety was covalently grafted onto the CO-releasing 3-hydroxyflavone (3-HF) derivatives, and the residue of 3-HF could act as a light-absorbing antenna to enable the corelease of CO and NO under 410 nm light irradiation. The compound subsequently self-assembled into a micelle, which exerted a synergistic antibacterial effect by CO and NO and efficiently killed *S*. *aureus*, outperforming the micelles that are capable of releasing CO or NO only. Moreover, this CO/NO releasing micelle also exhibited a higher antibacterial activity than vancomycin against MRSA-infected cutaneous wounds. In another study, Duan and coworkers developed an amphiphilic diblock copolymer poly(ethylene oxide)-*b*-poly(4-((2-nitro-5-(((2-nitrobenzyl)oxy)methoxy)benzyl)-(nitroso)amino)benzyl methacrylate) (PEO-*b*-PNNBM, termed PNOFA), which could release NO and formaldehyde (FA) upon 410 nm light irradiation (Fig. 5b) [166]. PNOFA could self-assemble into a micelle without premature gas leakage, while NO and FA could be released from the PNOFA micelle under 410 nm light to kill both *S*. *aureus* and *E*. *coli*. The above two studies both develop a photoresponsive micelle that can codeliver and corelease two kinds of antibacterial gases from a single donor, and reveal the advantages of the synergy of various gases in antibacterial treatment.

## 3 CO

**Fig. 6:**
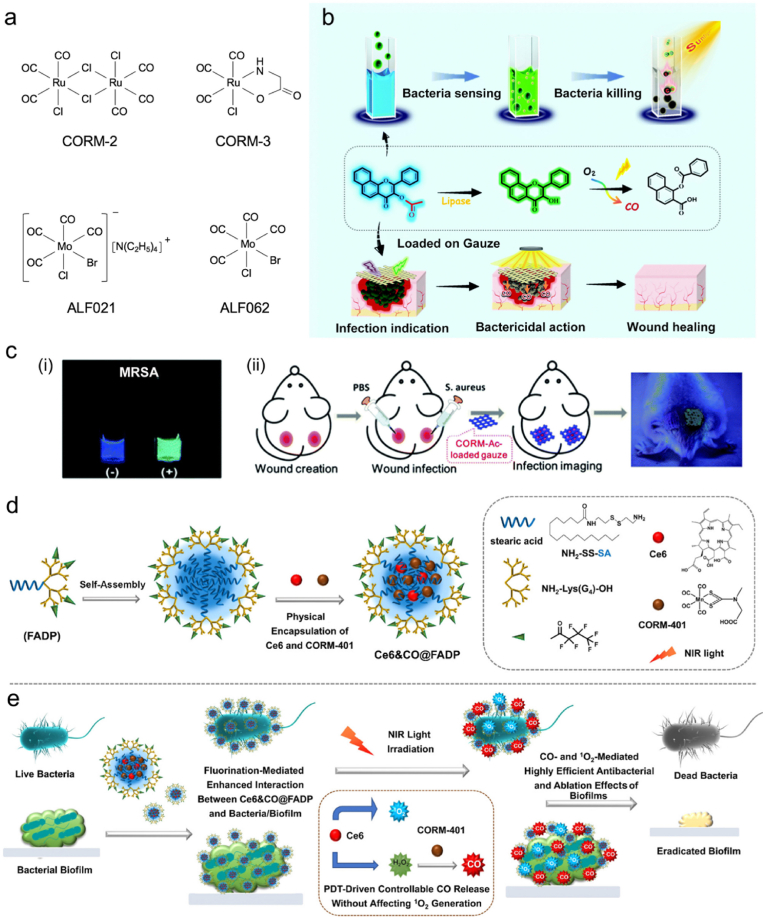
(a) Structures of CORM-2, CORM-3, ALF021, and ALF062. (b) Scheme showing the processes of bacterial sensing and subsequent bacterial killing in situ using CORM-Ac. (c) (i) Fluorescence change of CORM-Ac in the presence (+) or in the absence (−) of MRSA. (ii) Scheme showing the procedures of theranostic CORM-Ac-gauze for *S*. *aureus*-infected wound imaging. Reproduced with permission from Ref. [182]. Copyright 2020, Royal Society of Chemistry. (d) Schematic illustration of the preparation of Ce6&CO@FADP. (e) Proposed mechanisms of synergistic antibacterial and antibiofilm effect of Ce6&CO@FADP. Reproduced with permission from Ref. [183]. Copyright 2020, American Chemical Society.

CO can be produced by heme degradation via the catalysis of heme oxygenase (HO) enzymes in mammals, and it may exhibit physiological functions including antiinflammation and antiapoptosis [171,172]. During the past few decades, a number of studies have demonstrated the benefit of low-dose CO in antibacterial and antiinflammatory applications [173]. After entering bacteria, CO can bind to the terminal oxidases, competing with oxygen and inhibiting respiration to kill bacteria [49]. In addition, CO exhibits great stability at physiological pH, which allows it to exert therapeutic effects in distant sites [174]. Moreover, CO can also promote a key host defense mechanism—phagocytosis. When exposed to CO, bacteria can redistribute Toll-like receptor-4 (TLR-4) on the cell surface [175] and activate autophagy [176] or P2X7 receptor [177] to stimulate macrophage phagocytosis [178]. Till date, many CO donors have been developed for antibacterial uses (Table 2). However, the limited solubility of CO in water restricts its direct use in antibacterial applications. Therefore, three delivery approaches of CO have been developed: inhalation of gaseous CO, genes encoding HO enzymes, and utilization of CO-releasing molecules (CORMs) [173]. Among them, CORMs are considered as a convenient and safe way to deliver CO. Generally, CORMs are organometallic complexes, which release CO in an efficient and controlled way to reach high concentrations of CO [179]. In 2007, Nobre et al. evaluated the antibacterial effects of CORMs, including tricarbonyldichlororuthenium(II) dimer (CORM-2), tricarbonylchloro(glycinato)ruthenium(II) (CORM-3), bromo(pentacarbonyl)manganese (ALF021), and tetraethylammonium molybdenum pentacarbonyl bromide (ALF062) (Fig. 6a) [180]. Using *E*. *coli* and *S*. *aureus* as model bacterial strains, the authors demonstrated the remarkable antibacterial activity of CORMs under aerobic and anaerobic conditions. In a later study, Desmard et al. reported the bactericidal activities of CORM-2 and CORM-3 toward *P*. *aeruginosa* [181]. Therefore, gaseous CO or CORMs can act as effective and potential agents for antibacterial treatment.

### 3.1 Ruthenium-based CORMs

CORM-2 and CORM-3 are the most well-known ruthenium-based CORMs, which have been extensively used for antibacterial applications [184]. As early as 2002, Motterlini et al. reported a ruthenium-based CORM for the first time [185]. In 2010, Mann comprehensively reviewed the development of CORMs [186]. Nowadays, CORM-2 has been commercially available for a long time, while CORM-3 has only been marketed in recent years. Murray et al. utilized CORM-2 to control the growth of *P*. *aeruginosa* by killing bacteria within the formed biofilm and preventing biofilm maturation [187]. Similarly, CORM-2 was also reported by Bang et al. to fight against *E*. *coli* [188].

Tavares et al. reported that *Helicobacter pylori* (*H*. *pylori*) was susceptible to CORM-2 and CORM-3, and several metronidazole-resistant *H*. *pylori* clinical isolates could be killed by CORM-2 [189]. In addition, they found that combining sublethal doses of CORM-2 with metronidazole, clarithromycin, or amoxicillin could enhance the efficacy of the antibiotics. *In vivo* studies showed that CORM-2, either alone or combined with metronidazole, significantly reduced the possibility of *H*. *pylori* to infect animal cells. Sahlberg Bang and coworkers reported the antibacterial effect of CORM-2 on the biofilm of extended spectrum β-lactamase (ESBL)-producing uropathogenic *E*. *coli* (UPEC) [190]. After that, they assessed the transcriptomic impact of CORM-2 in a multidrug-resistant ESBL-producing UPEC isolate after single or repeated exposure to CORM-2 [191]. They found that repeated exposure to CORM-2 did not change the gene expression patterns and fold changes of ESBL-producing UPEC, and viability assays revealed the sustained susceptibility of ESBL-producing UPEC to CORM-2 after repeated exposure. This work demonstrates that CORM-2 can be repeatedly used for antibacterial therapy without the development of drug resistance.

In addition, several CORM-2-containing copolymers have been developed for antibacterial applications. For instance, Nguyen and coworkers reported CORM-2-conjugated thiodiblock copolymers for eradication of *P*. *aeruginosa* [192]. In another study, Maiti et al. fabricated a methionine methacryloyloxyethyl ester (METMA)- and poly(ethylene glycol methyl ether methacrylate) (PEGMA)-containing block copolymer, and then attached CORM-2 to the methionine side chain units to form P(METMA-*b*-PEGMA-CORM) for the prevention of *P*. *aeruginosa* biofilm formation [193].

Since CORM-2 is only soluble in dimethyl sulfoxide (DMSO), many researchers have adopted CORM-3 which is water-soluble in their studies. For instance, Rana et al. utilized CORM-3 to eradicate *Salmonella enterica* serovar Typhimurium (*S*. Typhimurium) [194]. The authors showed that CORM-3 was toxic to this bacterium at low concentrations (less than 100 μM) and the ruthenium was gradually accumulated to high levels intracellularly. Additionally, they demonstrated that CO could bind to the terminal oxidases of *S*. Typhimurium in situ to destroy its electron transport chain and kill it under physiological conditions. Besides, CORM-3 has also been used to fight against *P*. *aeruginosa* [195] and *E*. *coli* [196,197].

### 3.2 Photoactivated CORMs (photoCORMs)

[Mn(CO)_3_(tpa-κ^3^N)]Br is a kind of Mn-based photoCORMs, which can be triggered under light to realize precise and controllable release of CO. In 2014, Nagel et al. used [Mn(CO)_3_(tpa-κ^3^N)]Br to treat *E*. *coli* [198]. Photoactivation of [Mn(CO)_3_(tpa-κ^3^N)]Br at 365 nm could lead to the transfer of CO to the terminal oxidases of bacteria, giving rise to a pronounced growth inhibition effect toward *E*. *coli*. Tinajero-Trejo et al. also utilized [Mn(CO)_3_(tpa-κ^3^N)]Br as a photoCORM to kill an antibiotic-resistant uropathogenic strain of *E*. *coli* [199]. The growth and viability of *E*. *coli* were inhibited by activated [Mn(CO)_3_(tpa-κ^3^N)]Br while no effect was observed without photostimulation. Moreover, the authors found that the activated photoCORM could react with H_2_O_2_ to produce hydroxyl radical (•OH), further enhancing its toxicity. This work provides an approach of site-specific release of CO for antibacterial application. Betts et al. combined [Mn(CO)_3_(tpa-κ^3^N)]Br with colistin to fight against the multidrug-resistant strains of avian pathogenic *E*. *coli*, and a superb bacterial killing activity was observed [200].

Trypto-CORM is another photoCORM, and it usually exhibits toxicity against bacteria following photoactivation. Ward et al. used Trypto-CORM to kill *E*. *coli* under 400 nm light irradiation [201]. In addition, in 2017, they found that Trypto-CORM was toxic to *Neisseria gonorrhoeae* (*N*. *gonorrhoeae*) without light irradiation [202]. The authors reasoned that *N*. *gonorrhoeae* was more sensitive to CO-based toxicity than other bacterial pathogens, and thus a tiny amount of CO released from Trypto-CORM in the dark could cause damage to *N*. *gonorrhoeae*. This work suggests that CORMs can serve as specific antimicrobial agents against *N*. *gonorrhoeae*.

Different from directly using the already existing photoCORMs, Klinger-Strobel and coworkers noncovalently embedded Mn_2_(CO)_10_ into an electrospun poly(l-lactide-*co*-dl-lactide) nonwoven to release CO under 405 nm light irradiation against *S*. *aureus* biofilms [203]. This nonwoven exhibited a CO-induced antimicrobial activity, which reduced the number of bacteria within the biofilm by 70% after photostimulation. In another example, Mansour synthesized and characterized two new photoactivatable CORMs, *fac*-[Mn(CO)_3_(BZM)Br] and [RuCl_2_(BZM)(CO)_2_], derived from bromazepam (BZM, an anti-anxiety drug) [204]. Both of the CORMs could achieve CO release upon LED light irradiation, and thus showed remarkable antibacterial activity against *S*. *aureus* and *E*. *coli*. Recently, Wang et al. reported an enzyme-sensitive and photoactivatable CO-releasing platform for the successive detection and eradication of *S*. *aureus* and MRSA [182]. In this study, a CORM (3-hydroxy-2-phenyl-4*H*-benzo[*h*]chromen-4-one) was first synthesized by benzaldehyde and 1′-hydroxy-2′-acetonaphthone via the Algar–Flynn–Oyamada reaction, and it subsequently reacted with acetic anhydride to form *O*-acetyl group-protected CORM (CORM-Ac). The *O*-acetyl group in CORM-Ac could be enzymatically cleaved by the extracellular bacterial lipase to activate the excited state intramolecular proton transfer process, which could provide a visualized fluorescence signal to reveal the early infection (Fig. 6b). *In vitro* and *in vivo* experiments showed the remarkable changes in fluorescence color (Fig. 6c). After the change of fluorescence signal, CO could be released upon photoexcitation for antibacterial therapy. The validity and efficacy of CO in elimination of *S*. *aureus* and MRSA and dispersion of their biofilms were also demonstrated. This work develops a “sense-and-treat” platform for sensitive warning and effective treatment of bacterial infection.

### 3.3 Combination of CORMs and PDT, PTT, or antibiotic treatment

Antibacterial PDT and PTT have been considered to be powerful weapons to fight against bacterial infections, especially those caused by multidrug-resistant bacteria, because of their high photoactivation specificity and broad antibacterial spectrum, and they can be combined with not only NO therapy but also CO therapy. Inspired by this, Ma et al. designed a PDT-driven CO controllable release system for efficient ablation of biofilms formed by *S*. *aureus* and *E*. *coli* [183]. In aqueous solutions, the as-synthesized fluorinated amphiphilic dendritic peptide (FADP) molecules self-assembled into stable NPs, which provided hydrophobic cores for encapsulation of Ce6 and [Mn(CO)_4_{S_2_CNMe(CH_2_CO_2_H)}] (CORM-401) to obtain Ce6&CO@FADP (Fig. 6d). The fluorination of FADP was considered to enhance the interaction between Ce6&CO@FADP and bacteria and accelerate the endocytosis of Ce6&CO@FADP. After entering bacteria, Ce6&CO@FADP could generate massive ^1^O_2_ and H_2_O_2_ upon 665 nm NIR light irradiation, and the H_2_O_2_ could further oxidize CORM-401 to release CO (Fig. 6e). Consequently, the combination of ^1^O_2_ and CO exerted synergistic biofilm ablation effect *in vitro*. Besides, under NIR light irradiation, Ce6&CO@FADP significantly alleviated subcutaneous bacterial infection *in vivo*. Additionally, biosafety assessment results proved the satisfactory biocompatibility of Ce6&CO@FADP. In summary, Ce6&CO@FADP realized enhanced interaction with bacteria and fast endocytosis by virtue of the fluorination effect, and the produced ^1^O_2_ and released CO did not interfere with each other. Thus, this system achieves efficient and safe antibacterial treatment and provides a solution for the ablation of bacterial and biofilm infections in clinical use.

**Fig. 7:**
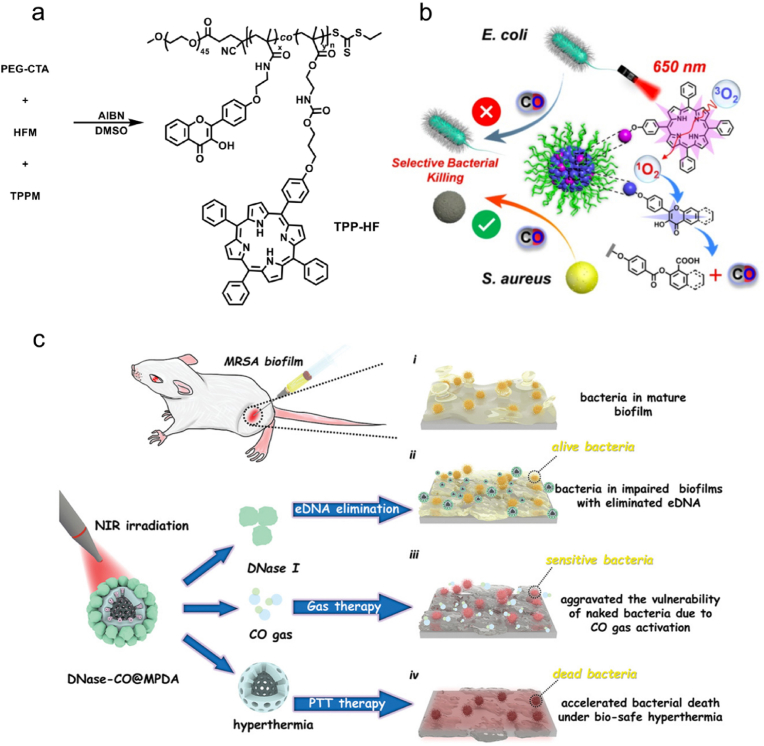
(a) Synthesis of the TPP-HF diblock copolymer. (b) Generation processes of ^1^O_2_ and CO under 650 nm light irradiation and selective bactericidal activity of TPP-HF toward Gram-positive bacteria. Reproduced with permission from Ref. [205]. Copyright 2021, Wiley-VCH. (c) Scheme illustrating the MRSA biofilm eradication through DNase I, CO gas, and hyperthermia. Reproduced with permission from Ref. [207]. Copyright 2021, Wiley-VCH.

Cheng et al. connected the 3-hydroxyflavone (3-HF)-based monomer (HFM) and the tetraphenylporphyrin (TPP)-based monomer (TPPM) via a PEG-based chain transfer agent (PEG-CTA) in the presence of AIBN to obtain TPP-HF diblock copolymer in DMSO (Fig. 7a) [205]. In aqueous solutions, TPP-HF could self-assemble to form micelles, wherein 3-HF and TPP were integrated into the cores. In this micellar system, TPP would be excited upon 650 nm light irradiation and convert ^3^O_2_ to ^1^O_2_, which could spontaneously oxidize 3-HF to generate CO (Fig. 7b). Unlike non-specific antibacterial agents, this CO-releasing micelle could be selectively internalized by *S*. *aureus* instead of *E*. *coli* and exert a selective bactericidal effect. Moreover, it was demonstrated that the micelles could simultaneously cure MRSA-infected wounds, eradicate MRSA pathogens, and accelerate wound healing. This study provides a strategy to activate photoCORMs for selective elimination of *S*. *aureus* and MRSA-infected wound treatment. Very recently, Zhou et al. developed an antibacterial PDT nanoplatform (termed POS-UCNPs/ICG) consisting of two-dimensional (2D) partially oxidized tin disulfide (SnS_2_) nanosheets (POS NSs), UCNPs (NaYF4:20%Yb^3+^,2%Er^3+^@NaYF4:20%Yb^3+^,30%Nd^3+^ core–shell NPs), and ICG [206]. The POS NSs were demonstrated to yield CO and O_2_ by visible light irradiation. Additionally, UCNPs could transform 808 nm light to green light, which further facilitated the reduction of CO_2_ and oxidization of H_2_O to produce CO and O_2_. With the same 808 nm excitation light, ICG could produce plenty of ROS with the O_2_ supply. Therefore, POS-UCNPs/ICG could realize antibacterial PDT to effectively inhibit *S*. *aureus* and *E*. *coli*.

Yuan et al. developed NIR light-activatable DNase–CO@MPDA (DNase is the abbreviation of deoxyribonuclease) NPs to eliminate MRSA and alleviate inflammatory responses in MRSA biofilm-infected wounds [207]. In this work, thermosensitive CO-releasing donors (Fe_3_(CO)_12_) were first loaded into MPDA NPs, followed by covalently immobilizing DNase I on the surfaces of MPDA via michael addition. DNase I could degrade the extracellular DNA (eDNA) in biofilms and then site-specifically destroy the compactness of the biofilms. Under NIR light irradiation, the DNase–CO@MPDA NPs exhibited excellent photothermal performance and the temperature rise further triggered the release of CO to permeate through the impaired biofilms. Eventually, the synergistic effects of DNase I, CO, and MPDA achieved effective MRSA biofilm elimination (Fig. 7c).

Additionally, De La Cruz et al. combined CORM with an antibiotic to kill *H*. *pylori* [208]. They synthesized a small-molecule donor to codeliver and release CO and the antibiotic metronidazole. Notably, the MIC values against *H*. *pylori* decreased from 2.5 μg mL^−1^ for metronidazole alone to 0.31 μg mL^−1^ for the CO- and metronidazole-releasing donor. This work highlights that the combined use of CO gas and an antibiotic can achieve significantly improved antibacterial effect.

## 4 SO_2_

SO_2_, besides being an environmental pollutant, is also recognized as an endogenous gasotransmitter with the same importance as that of NO, CO, and H_2_S [46,209]. Endogenous SO_2_ gas plays an important physiological role in regulating blood vessel and cardiac function at low concentrations [210]. However, SO_2_ can damage biomacromolecules at high concentrations [51]. It can also induce various modes of cellular stresses such as perturbation of redox homeostasis. For instance, plenty of radical species (SO_3_^•–^, SO_4_^•–^, or SO_5_^•–^) can be generated during the auto-oxidation reaction of the hydrated forms of SO_2_ and sulfite to sulfate to damage DNA [211]. In addition, the widespread use of sulfites as preservatives in food industry also indicates their capacity to destroy microbes [50]. Thus, introduction of SO_2_ intracellularly may cause irreversible perturbation to the redox equilibrium because the resultant oxidative stress is difficult for the corresponding pathogen to overcome [212,213], and delivering SO_2_ to infection sites may have enormous potential in antibacterial applications.

**Fig. 8:**
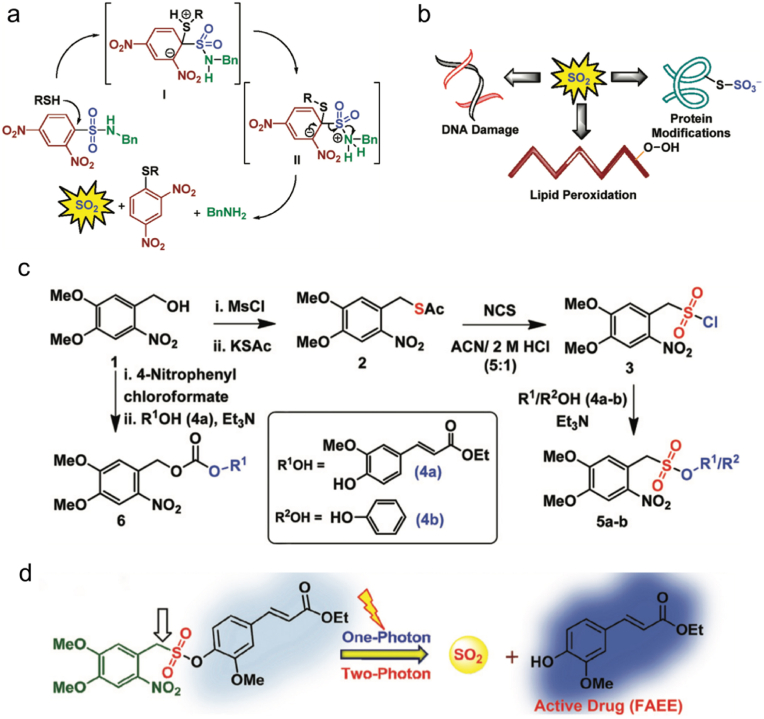
(a) Schematic illustration of the mechanism of thiol-mediated SO_2_ generation. (b) Possible biological targets of SO_2_. Reproduced with permission from Ref. [215]. Copyright 2012, American Chemical Society. (c) Synthetic routes of SO_2_ donors 5a, 5b, and carbonate donor 6. (d) Scheme of the proposed one- and two-photon activated phototrigger for the release of SO_2_ and an FAEE. Reproduced with permission from Ref. [217]. Copyright 2019, Royal Society of Chemistry.

Murano et al. reported the antibacterial efficacy of sodium bisulfite (NaHSO_3_) against *H*. *pylori* [214]. NaHSO_3_ was ionized in solution and supplied SO_2_, which entered bacterial cells and then inhibited ATP synthesis and bacterial metabolism to kill bacteria. Malwal et al. synthesized 2,4-dinitrophenylsulfonamide, a thiol-activated SO_2_ donor, to fight against *Mycobacterium tuberculosis* (*Mtb*) [215]. *Mtb* contains mycothiol (MSH) as the primary thiol (RSH), and such RSH could react with 2,4-dinitrophenylsulfonamide to produce intermediate I, which was further converted to intermediate II through a proton transfer process. Then, the collapse of intermediate II produced benzylamine (BnNH_2_), 2,4-dinitrophenylthioether, and SO_2_ (Fig. 8a). Of note, MSH plays a crucial role in maintaining the redox homeostasis of *Mtb*. Hence, the reaction between MSH and 2,4-dinitrophenylsulfonamide could lead to the decrease of the thiol level of *Mtb* and induce the stress or death of *Mtb*. In addition, the generated SO_2_ could further affect cellular redox equilibrium and cause damage to biomacromolecules, including DNA, lipids, and proteins (Fig. 8b). Later, the same group utilized 2,4-dinitrophenylsulfonamide to treat MRSA, and a remarkable antibacterial efficacy was achieved [216].

Venkatesh et al. fabricated a light-activatable SO_2_ donor based on the 4,5-dimethoxy-2-nitrobenzyl (DMNB) phototrigger to inhibit *Enterobacter cloacae* (*E*. *cloacae*) [217]. In this study, three donors were first synthesized as follows. As shown in Fig. 8c, the DMNB alcohol 1 was treated with mesyl chloride and potassium thioacetate to obtain the intermediate thioacetate 2, which was subsequently treated with *N*-chlorosuccinamide to form sulfonyl chloride 3. The as-synthesized sulfonyl chloride further reacted with ferulic acid ethyl ester (FAEE, 4a) or phenol (4b) to obtain the SO_2_ donor 5a or 5b. Besides, a carbonate donor 6 was prepared by treatment of the DMNB alcohol 1 with 4-nitrophenyl chloroformate, followed with the addition of 4a. After evaluating the antibacterial activities of these three donors, the authors found that the SO_2_ donor 5a exhibited an enhanced inhibitory effect against *E*. *cloacae* compared with donor 5b and donor 6. It was considered that the superior antibacterial effect of donor 5a was ascribed to its dual release of FAEE, a hydroxyl-based drug with broad antibacterial activity, and SO_2_. Before photolysis, donor 5a showed no remarkable inhibitory activity toward *E*. *cloacae*. However, the donor 5a could generate SO_2_ along with FAEE after one- and two-photon activation (Fig. 8d) and showed a high inhibitory activity toward *E*. *cloacae* in 1 h. Additionally, the authors evaluated the antibacterial activity of FAEE alone, which was weaker than that of the combination of FAEE and SO_2_. This work achieves controlled release of SO_2_ via light illumination, and the synergy of SO_2_ and FAEE exhibits remarkable antibacterial activity.

## 5 H_2_S

H_2_S is recognized as a gasotransmitter in mammals and participates in multiple physiological processes [218]. In most mammals and some bacteria, H_2_S is produced by three enzymes: cystathionine γ-lyase, cystathionine β-synthase, and 3-mercaptopyruvate sulfurtransferase [219]. In the past few years, H_2_S has been found to possess many beneficial functions. In particular, H_2_S has the capacity to scavenge ROS, thus preventing cells from oxidative stress [220]. In 2011, Shatalin et al. first proposed an antibiotic resistance mechanism mediated by H_2_S for several pathogenic bacteria [52]. They proposed that H_2_S could mitigate the oxidative stress imposed by antibiotics or reduce intracellular ROS level to protect bacterial cells. In 2018, however, Weikum et al. reported the impact of exogenously added sulfide on the physiology of *S*. *aureus* [53]. Results showed that the sulfide could only protect *S*. *aureus* from aminoglycoside antibiotics, while exogenous introduction of sulfide could exacerbate the elimination of *S*. *aureus* by folic acid inhibitors, glyco(lipo)peptides, and other kinds of antibiotics, including tetracyclines, quinolones, and β-lactams. In addition, *S*. *aureus* was found to be unable to produce a substantial amount of sulfide. Therefore, the authors suggested that the protection of sulfide produced by bacteria against antibiotics should not be regarded as a universal defense mechanism against antibiotics, and sulfide, such as H_2_S, could act as an alternative antibacterial agent against the bacteria that can only endogenously produce a small amount of H_2_S (e.g., *S*. *aureus*) or those that are unable to produce endogenous H_2_S. In recent years, many researchers have demonstrated the high toxicity of H_2_S to bacteria, and they reported that H_2_S can significantly inhibit the growth of *S*. *aureus*, *E*. *coli* [53], *Aspergillus niger*, *Penicillium italicum* [54], as well as several marine bacteria [55].

Fu et al. utilized sodium hydrosulfide (NaHS, a H_2_S donor) to investigate the effect of H_2_S on the growth of *E*. *coli* [56]. They reported that H_2_S treatment decreased GSH level and stimulated the production of ROS in *E*. *coli*, giving rise to lipid peroxidation and DNA damage. In another example, Wu and coworkers reported synergistic effects of exogenous H_2_S and H_2_O_2_ on *Shewanella oneidensis* (*S*. *oneidensis*) [57]. H_2_S could inactivate KatB, a heme-containing enzyme implicated in H_2_O_2_ degradation, which promoted the toxicity of H_2_O_2_ toward *S*. *oneidensis*. After that, Ng et al. utilized NaHS as an H_2_S-releasing agent and investigated the effect of exogenous H_2_S on *Acinetobacter baumannii* (*A*. *baumannii*), a kind of non-endogenous-H_2_S producing bacteria [58]. The exogenous H_2_S triggered a pro-oxidative redox disbalance of *A*. *baumannii* and made this bacterium be sensitive to antibiotics like GEN, colistin, rifampicin, and clarithromycin. Therefore, the combined use of H_2_S and antibiotics enhanced the bacterial killing performance of these antibiotics. Moreover, the GEN-resistant *A*. *baumannii* was treated with GEN and NaHS alone or combined, and the results indicated that NaHS weakened the bacterial resistance to GEN, thus enhancing the antibacterial efficacy of GEN. This work proves that H_2_S-releasing compounds can be used as resistance-reversion agents and antibiotic-potentiators to fight against bacteria that do not produce endogenous H_2_S.

In another study, Aslami and coworkers infected rats with *Streptococcus pneumoniae* (*S*. *pneumoniae*) to construct a rat model of pneumococcal pneumosepsis and then intravenously infused NaHS to the rats [59]. On the one hand, H_2_S produced by the infused NaHS kept the bacterial outgrowth unchanged. On the other hand, NaHS treatment was proved to lead to the increase of the transcription of mitochondrial respiratory subunits and subsequently decrease the oxidative phosphorylation and promote ATP synthesis. These processes could stabilize bio-energetic status and thus protect local and distant organs from being infected by bacteria and reduce their injury in the pneumosepsis model. This work displays the dual functions of H_2_S and develops a potential therapeutic approach to mitigate pneumosepsis in clinical use.

## 6 H_2_

Hydrogen (H_2_) is recognized as a reductant to react with highly reactive oxidants—including ONOO^–^ and •OH—in mammalian cells [221]. It is reported that H_2_ can scavenge intracellular ROS without any toxic effect even at high concentrations [222]. H_2_ has several advantages in medical applications. First, H_2_ is mild enough so that it will not affect ROS functioning in cell signaling or disturb metabolic redox reactions. Second, H_2_ is the only antioxidant which can cross the blood-ocular and blood-brain barriers [223]. Third, H_2_ also displays antiapoptotic, antiinflammatory, cytoprotective, and mitohormetic properties. Recently, H_2_-mediated treatment has been proved to be effective and promising in antibacterial uses.

**Fig. 9:**
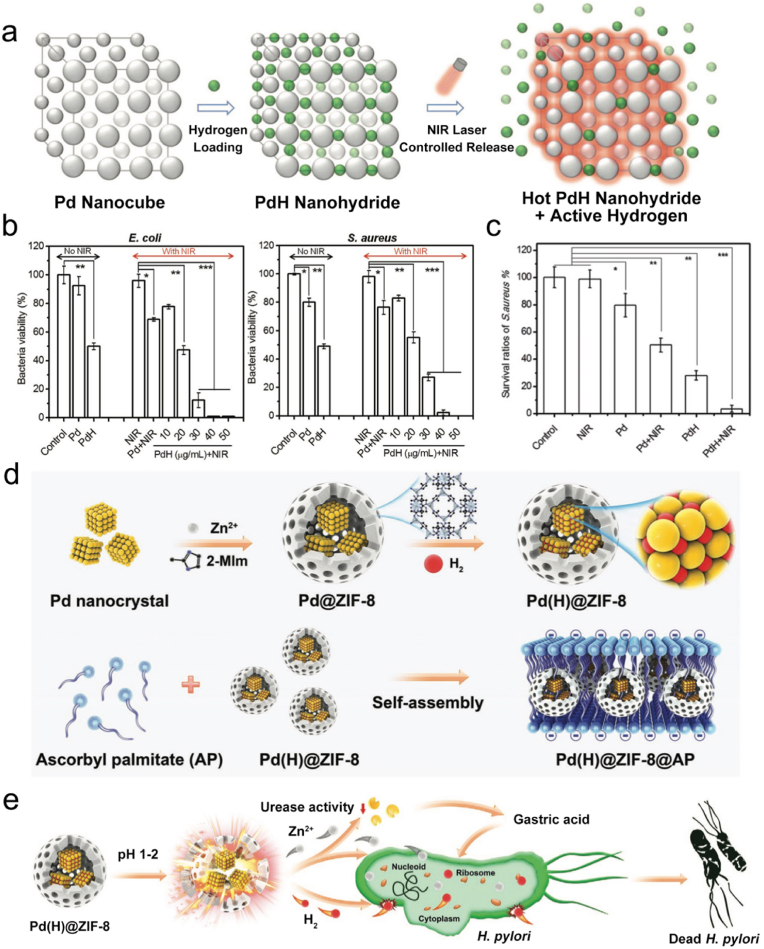
(a) Scheme showing the synthetic process of PdH nanohydride and its NIR laser-controlled H_2_ release property. (b) Relative viabilities of *E*. *coli* and *S*. *aureus* with different treatments. (c) Survival ratios of *S*. *aureus* in rat's wounds in different groups. Reproduced with permission from Ref. [60]. Copyright 2019, Wiley-VCH. (d) Scheme depicting the preparation processes of Pd(H)@ZIF-8 and Pd(H)@ZIF-8@AP. (e) Scheme showing the antibacterial mechanism of Pd(H)@ZIF-8 under the gastric acid condition. Reproduced with permission from Ref. [61]. Copyright 2021, Wiley-VCH.

Yu and coworkers first synthesized a Pd nanocube with the ability to absorb and release H_2_ at normal pressure and temperature, and then incorporated H_2_ into the nanocube to obtain a hydrogen-releasing PdH nanohydride for antibacterial treatment and bacteria-infected wound treatment (Fig. 9a) [60]. This nanohydride combined photothermal effect of Pd and antibacterial effect of bioactive H_2_. Upon 808 nm NIR laser irradiation, the Pd–H binding force was destroyed, giving rise to the temperature rise of Pd nanocube and the release of active H_2_. *In vitro* experiments demonstrated a strong bacteria-killing capacity of the PdH nanohydride toward *E*. *coli* and *S*. *aureus*, and with irradiation of NIR laser, almost no bacteria were found when the PdH concentration was over 40 μg mL^−1^ (Fig. 9b). Moreover, the efficacy of PdH nanohydride for treating *S*. *aureus*-infected wounds of rats was also assessed, and the combinational hydrogen-photothermal treatment exhibited a remarkable bactericidal effect *in vivo* and was effective in wound healing. The survival ratio of *S*. *aureus* in the rats’ wounds of different groups was evaluated after 10-day treatment, and almost all the *S*. *aureus* bacteria in wounds were eliminated (Fig. 9c). Later, Zhang et al. designed a pH-responsive H_2_-generation platform to kill *H*. *pylori* and restore impaired gastric mucosa [61]. In this work, Pd NPs were first loaded into zinc-based ZIF-8 to form Pd@ZIF-8, into which H_2_ was introduced to obtain Pd(H)@ZIF-8 due to the specific interaction between Pd NP and H_2_. Subsequently, Pd(H)@ZIF-8 was encapsulated into negatively charged ascorbate palmitate (AP) hydrogel to obtain Pd(H)@ZIF-8@AP (Fig. 9d). It has been proved that the outer AP hydrogel could target positively charged inflammatory sites and be hydrolyzed by matrix metalloproteinase (MMP) which is enriched in inflammatory sites. Then, the exposed Pd(H)@ZIF-8 could be decomposed by gastric acid to release H_2_ and Zn^2+^. H_2_ disrupted the permeability of *H*. *pylori* to facilitate the entry of Zn^2+^ into the cells, leading to the leakage of cell content and interfering with cell metabolism. Moreover, Zn^2+^ could inhibit the activity of urease to enhance the invasion of gastric acid on *H*. *pylori*, thus achieving satisfactory antibacterial performance (Fig. 9e). In addition, the released H_2_ could scavenge redundant oxygen free radicals to protect gastric epithelial cells from being destroyed by oxidative stress. It was also found that H_2_ could regulate the secretion of inflammatory factors from macrophages and promote the expression of mucosal repair proteins, thus alleviating hyperactive inflammatory response and restoring impaired gastric mucosa. In summary, this platform has multiple functions including antibacterial capacity, inflammatory regulation ability, as well as repair function of damaged gastric mucosa, and represents a precise and effective approach for *H*. *pylori* treatment.

## 7 Conclusions and perspectives

In this review, we carefully summarize the strategies and advances of antibacterial gas therapy in the past decade. These gases cover NO, CO, SO_2_, H_2_S, and H_2_, which are recognized as double-edged molecules. On the one hand, they are essential signaling molecules involved in many important physiological processes in mammalian cells and are crucial for maintaining tissue homeostasis at low concentrations. On the other hand, they can damage biomacromolecules or even cause cell death at high concentrations. Numerous studies have reported that these gases display potent antibacterial effects *in vitro* and in skin infection models when used at appropriate concentrations, while most of the normal cells and the surrounding tissues appear to be unaffected by the exposure to these gases.

Given the potential bacterial resistance and extended treatment period of traditional antibiotics, it is desirable to find more promising antibacterial strategies. Gas therapy has been proved to reduce the likelihood of resistance issues and may provide an alternative solution for antibacterial applications. As a result, many efforts have been made to design gas-releasing materials for antibacterial treatments. For instance, some studies suggest that NO can enhance the bactericidal effect due to its capacity to realize deeper penetration within bacteria-infected sites and dispersion of biofilms without developing resistance. Moreover, the integration of these gases with PDT, PTT, or antibiotic treatment can minimize the required amount of antibiotics or reduce the required concentration of bactericidal materials to treat different bacteria. Despite its rapid development and encouraging performance, antibacterial gas therapy still face some challenges that need to be overcome in the future.

First, the biological mechanisms regarding the antibacterial performance of different gas molecules need to be further explored. So far, only a few specific mechanisms have been figured out. For example, NO has been proved to cause nitrosative stress in bacteria or alter the level of c-di-GMP to disperse biofilms, while CO can compete with oxygen and bind to the terminal oxidase active site of respiratory chain in bacterial cells, thus inhibiting respiration to kill bacteria. H_2_ is considered to disrupt the permeability of bacterial membrane to facilitate the entry of antibiotics or other drugs. However, the specific action mechanisms of other gases, such as SO_2_ and H_2_S, in antibacterial treatment still remain elusive up till now. Some studies inferred that the antibacterial effect of SO_2_ may be caused by oxidative stress, while the antibacterial mechanism of H_2_S is still unclear. Additionally, the appropriate therapeutic doses of different gases required for different bacteria or bacterial infection models are unclear and difficult to determine at present. Accordingly, in-depth mechanistic investigations are necessary to realize accurate and efficient antibacterial treatments. Moreover, other gases, including O_2_, carbon dioxide (CO_2_), nitrogen (N_2_), and perfluorocarbon, have exhibited their anticancer effect [224]. We expect that these gases may also have the potential for antibacterial applications to some extent. Therefore, researchers should be dedicated to exploring and utilizing the antibacterial activities of the above-mentioned gases and others in future studies.

Second, diverse gas donors and more controllable gas delivery and release systems should be developed to optimize the therapeutic efficacy and reduce side effects to normal cells and tissues. Many of the current gas-releasing systems are designed to slowly and continuously release gas molecules, resulting in the poor controllability of the gas release rate. In the future, more gas-releasing systems should be developed to respond to endogenous or exogenous stimuli such as bacterial secretions, NIR light, heat, ultrasound, and electromagnetic field. What is more, it is difficult to control the amount of gas released from these gas-releasing systems. An appropriate amount of released gas is crucial and necessary in antibacterial gas treatment, because supra-physiological levels of these gases can be toxic to surrounding cells and tissues, while low concentrations may protect the infected sites from oxidative stress or antibiotics.

Third, the antibacterial efficacy may be affected by the size and surface charge of the gas delivery system. In 2011, Carpenter et al. evaluated the relationship between the bactericidal effect and the size of an NO-releasing silica NP [225]. It was found that the smaller NP exhibited a higher bactericidal activity. One possible explanation is that a smaller size may contribute to an increased association rate between bacteria and NPs, thus leading to a larger gas payload delivered to the bacteria. Moreover, the association rate and the antibacterial effect are likely influenced by the surface charge of each system because bacteria usually exhibit a net negative charge—positively charged systems may more easily associate with bacteria compared with the systems with neutral and negative charges, and thus the gaseous molecules can be released close to the bacteria. Collectively, by figuring out the interactions between bacteria and gas delivery systems, more efficient gas delivery and stronger antibacterial effect can be realized.

Forth, compared with the use of traditional antibiotics alone, the combination of gaseous molecules and antibiotics can exhibit increased antibacterial activities. Such combination may minimize the amount of antibiotic usage or reduce the concentrations required to treat antibiotic-resistant “superbugs”. Moreover, the current antibacterial gas therapy is mainly combined with PDT or PTT. In the future, more therapies should be introduced and integrated with gas therapy to achieve better antibacterial effect.

Last but not least, to promote their clinical translation, the various gas generators developed should be subjected to careful biosafety evaluations. Although these gases are biocompatible and safe at suitable concentrations, the nanocarriers may have biosafety problems. For instance, for most of the commonly used inorganic CORMs, although they are relatively stable, they may cause potential toxicity to normal cells, tissues, or organisms because of the presence of metal elements. In addition, for organic gas-releasing systems, although many of them are biocompatible and biodegradable, they are highly unstable in complex and harsh microenvironments, resulting in uncontrollable gas release. Organic–inorganic hybrid systems can integrate the advantages and minimize the shortcomings of organic and inorganic materials, which may help to solve the biosafety issue. Nevertheless, although some of the developed systems have been proved to be biocompatible, they have complicated preparation procedures, which may limit their practical use. Before introducing these antibacterial gases—NO, CO, SO_2_, H_2_S, and H_2_—into preclinical and clinical trials and applications, the cyto/hemocompatibility, biodistribution, metabolism, and long-term biosafety need to be systematically and comprehensively evaluated.

We are entering the post-antibiotic era where enhancing antibacterial properties and overcoming antibiotic resistance are highly desirable. Fortunately, gas therapy just right meets the requirement of current antibacterial treatment despite the fact that antibacterial gas therapy is still at an early stage. We believe that continuous efforts put into gas-involved antibacterial treatment will boost the development of this emerging field, and gas therapy will act as a feasible and potent weapon to fight against bacterial infections and realize more clinic uses in the near future.

## Ethics approval and consent to participate

This is a review article and does not include any animal or human experiments.

## CRediT authorship contribution statement

**Tian-Yu Wang:** Investigation, Writing – original draft, Writing – review & editing. **Xiao-Yu Zhu:** Investigation, Writing – original draft, Writing – review & editing. **Fu-Gen Wu:** Conceptualization, Writing – review & editing, Supervision, Funding acquisition.

## Declaration of competing interest

The authors declare that they have no known competing financial interests or personal relationships that could have appeared to influence the work reported in this paper.
